# The configuration, funding and costs of providing services for children with behaviours that challenge. A report undertaken as part of the Mapping and Evaluating Services for Children with Learning Disabilities and Behaviours that Challenge (MELD)

**DOI:** 10.3310/nihropenres.14153.1

**Published:** 2026-02-11

**Authors:** Elizabeth-Ann Schroeder, Richard Hastings, Nicholas Manktelow, Ashley Liew, Mark Lovel, Caroline Richards, Jill Bradshaw, Nick Gore, Paul Thompson, Peter Langdon, Stavros Petrou, MELD Research Study Team

**Affiliations:** 1 Nuffield Department of Primary Care Health Sciences, University of Oxford, Oxford, England, OX26GG, UK; 2 School of Social Policy and Society, University of Birmingham, Birmingham, England, B15 2TT, UK; 3 Paediatric Liaison Psychiatry Service | National & Specialist CAMHS, South London and Maudsley NHS Foundation Trust, London, SE5 8AZ, UK; 4 Child & Adolescent Mental Health Service, Tees, Esk and Wear Valleys NHS Foundation Trust, Stockton on Tees, TS18 3TX, UK; 5 School of Psychology, University of Birmingham, Birmingham, England, B15 2TT, UK; 6 Tizard Centre, University of Kent School of Social Policy Sociology and Social Research, Canterbury, England, CT2 7LR, UK

**Keywords:** Intellectual disability, Behaviours that challenge, Children and young people, Community-based services, Service models, Health economics, Cost analysis

## Abstract

**Background:**

Children with intellectual disabilities and behaviours that challenge (BtC) and their families require complex, multi-agency support. Despite policy commitments to strengthen community-based provision, including those arising from Transforming Care and the forthcoming Mental Health Bill (2025), there remains limited evidence on how these services are configured or what they cost to deliver. The National Institute for Health and Care Excellence ( NICE, 2018) identified major evidence gaps in optimal service design, integration, and cost-effectiveness.

**Objective:**

This research explores the structure and associated costs of community-based services in England that support children with intellectual disabilities and BtC. It aims to describe the configuration of local “service models”, identify cost drivers, and clarify data requirements for commissioning and evaluation.

**Methods:**

Within the NIHR-funded MELD (Mapping and Evaluating Services for Children with Learning Disabilities and Behaviours that Challenge) programme, 278 services were identified across England. Using survey data (n=161), Latent Class Analysis and descriptive synthesis produced a typology of five community service models. Nineteen representative sites participated in a detailed costing study using a bespoke data collection instrument aligned with national unit-cost frameworks. Quantitative and narrative data on staffing, pathways, and overheads were analysed descriptively and thematically, with input from a Parent Advisory Group.

**Results:**

Five distinct service models were identified: generic CAMHS, ID-CAMHS, Children and Young People’s Disability, Specialist BtC Support, and All-Age services. Costs were primarily driven by staffing mix, intensity of BtC support, and organisational structure. Considerable heterogeneity was observed in funding flows, eligibility, and waiting times.

**Conclusions:**

This study provides the first national overview of community service configurations for children with intellectual disabilities and BtC. The findings highlight significant variation in organisation and cost, informing future economic evaluation and service-model optimisation in line with ongoing policy reform.

## Plain English Summary

This research investigated how services for children with intellectual disabilities and specifically, behaviours that challenge (BTC) in England are structured, and the associated costs of providing care. The study found that there is a gap in information about how these services are currently designed, what they cost, and how effective they are.

This research, the first of its kind, aimed to explore what data could be collected. Researchers found it difficult to get accurate cost information from services. They were often missing key details such as overheads and operational costs, and information about the children who are served. It turns out that a lot of data is either unavailable, or not recorded in a way that makes it easy to share.

Despite a national policy approach to shift care for people with intellectual disabilities and autism from hospitals to community settings, there are still major gaps. Many services have long waiting lists, suggesting that they are underfunded and unable to meet the high demand. This can lead to poorer health outcomes for children and significant stress for their families.

The report recommends that decision-makers who commission services for children with BTC, would be helped if services collected a basic set of data on these services, including details on staffing, funding, and the types of patients. This would help to streamline and connect services across different regions and to ensure funding is used effectively. It also suggests that future research should focus on a more person-centered approach to funding, considering the full range of a child’s needs and the costs borne by their families. Ultimately, the study highlights a critical need for better data collection to improve the quality of care for these children and their families.

**The Challenge of Data Collection:**

A central finding of the research was the immense difficulty in collecting accurate and complete data from these services. Researchers found that key cost information, such as operational expenses, overheads, and the specific needs of the children being served was often missing or incomplete. Frontline staff, already overworked, had little capacity to respond to data requests, and the data that did exist was often not readily available or was so integrated with other services that it was hard to separate. This lack of a standardized minimum dataset makes it incredibly difficult to assess the services' value for money or to plan for future needs.

**Policy Context and Systemic Issues:**

The study is set against a backdrop of UK policy changes, including the Transforming Care program and recent reforms to the Mental Health Act. These initiatives aim to shift care for people with intellectual disabilities and autism from hospitals to community settings. However, the research found that current community services are struggling ; many have significantly long waiting lists, suggesting they are underfunded and lack the capacity to meet demand. These delays can lead to worsening health for the children and immense stress for their families, who report poor experiences with the system.

**Recommendations and Future Directions:**

The report makes several key recommendations for commissioners and policymakers:

- Implement a Minimum Dataset: Start collecting a standardized set of data on funding, staffing, patient demographics, and service coverage to improve oversight and planning.
- Enhance Service Coordination: Streamline services across geographic areas to better coordinate care and guide families to the most appropriate support based on their child's needs.
- Adopt a Person-Centered Approach: Design services that prioritize early intervention and personalized care plans, which may require a different funding model that considers the full spectrum of a child's needs, including social and educational support.
- Measure Outcomes: Future research should go beyond just costs and measure the long-term outcomes for both the children and their families. This will help determine which service models are most effective and provide the best value.

In conclusion, this research highlights a critical and persistent gap between policy goals and the reality of service delivery. It underscores the urgent need for better data collection to ensure that community-based care for children with intellectual disabilities and challenging behaviours is not only well-intentioned but also effective and equitable.

## Background

This research explores the configuration and associated costs of community-based services in England that support children with intellectual disabilities and behaviours that challenge (BtC), and their families. The structure and organisation of these health and care services across localised areas are described as “service models”. The National Institute of Health and Care Excellence (NICE) identified evidence gaps about how best to design and deliver these services ( NICE, 2018), and a paucity of evidence also exists about the characteristics of the service models that are currently delivered, and their associated costs ( Taylor *et al.*, 2023). Given the lack of evidence available to inform ongoing planning and delivery, this research is the first of its kind; and provides an exploratory narrative in discovering what types of data can be collected and how to interpret it.

An ‘intellectual disability’ is a condition defined by low cognitive ability combined with low levels of adaptive behaviour, and it is classified as a disorder of intellectual development within the International Classification of Diseases ( Salvador-Carulla *et al.*, 2011). Some reports frequently use the term learning disability and others the term intellectual disability. For consistency in this report, we refer to learning disability as intellectual disability. It is estimated that in the UK, a subset of approximately twenty-percent of children with intellectual disabilities also display behaviours that challenge (BtC). BtC are a group of behaviours, such as aggression or self-injury, that whilst not attracting a medical diagnosis, may cause the child, carers or others to be at risk of harm, or may result in community exclusion ( Emerson & Einfeld, 2011). The prevalence of BtC is broadly similar across areas of the UK, though it varies slightly with socio-economic factors. Families of children with BtC are reported to experience greater emotional stress and reduced quality of life ( Hastings, 2016), and the services provided for the children are costly ( Einfeld *et al.*, 2010; Iemmi *et al.*, 2016). Previous research has also highlighted family carers’ reports of poor experiences with services including stressful interactions ( Griffith & Hastings, 2014).

In terms of the broader policy landscape, ‘Transforming Care’ was a national programme launched in the UK in 2012 to reduce the number of people with intellectual disabilities and/or autism as hospital inpatients into more appropriate, community-based care ( Department of Health, 2012). The programme initiated the (i) creation and expansion of specialized community teams to provide intensive support and crisis intervention, to reduce escalations to hospital admission, (ii) supported living services to help people live in their own homes with the right level of personal support and (iii) the creation of service directories and care pathways to enable families and professionals to find the right support and navigate the care system. The resultant principles and goals developed through Transforming Care have been a driver for more recent policies, including proposals to reform the Mental Health Act ( Department of Health and Social Care, 2021).

Significant policy reforms are underway, and the most recent development is the Mental Health Bill ( The Stationery Office, 2025). The Bill reinforces NICE’s earlier recommendations, noting that effective community-based services and support for BtC were a national priority ( NICE, 2015) requiring optimal service model design and organisation (NICE guidelines for BtC ( 2015; 2018)). An independent review then published in 2018 and a subsequent Government White Paper *Reforming the Mental Health Act* in 2021, led to a draft piece of legislation that is currently making its way through Parliament ( The Stationery Office, 2025). The planned changes to the Mental Health Act include an intention to prevent the inappropriate admission of people with intellectual disabilities and autism into inpatient units. These reforms are in response to concerns that individuals have been detained for extended periods in hospital settings that are not equipped to meet their needs, often for behaviours that are a result of their condition rather than a co-occurring mental illness ( Department of Health and Social Care, 2021).

The proposed legislation aims to shift care from inpatient settings to the community to promote a less restrictive approach and crucially, a greater emphasis on community support and services as the alternative. Without these, there is a risk that the reforms could fail to prevent inappropriate hospital admissions and detentions, and over-medication ( Department of Health and Social Care, 2021; NHS Confederation, 2024; The Centre for Mental Health, 2024). In policy alignment, the STOMP (Stopping the Over-Medication of People with an intellectual disability, autism or both) policy was launched by NHS England in 2016 ( NHS England, 2016). STAMP (Supporting Treatment and Appropriate Medication in Paediatrics) was then launched in 2018 to extend the same principles to children and young people ( NHS England, 2018). Both initiatives were key commitments within the NHS Long Term Plan ( NHS England, 2019). They aimed to address the issue of the (i) over-prescription of psychotropic medications to people with intellectual disabilities and/or autism to improve their quality of life by ensuring they are not prescribed medication (such as antipsychotics, antidepressants, and sedatives that are used to manage challenging behaviours) inappropriately (ii) to ensure medication is used only when clinically necessary and in conjunction with other non-pharmacological interventions, such as behavioural therapies, psychological support, and social care and (ii) to minimize the serious side effects associated with long-term use, such as weight gain, drowsiness, and an increased risk of other health problems ( NHS England, 2019)

The provision of community-based programmes, therapy, and support that are specifically tailored to the needs of people with intellectual disabilities and autism are now a political imperative ( Department of Health and Social Care, 2022). Recommendations by NICE (2018) support research to investigate service model attributes such as person-centred care, service integration, family support and the professional skills and competencies needed to deliver positive outcomes. The research presented here explores the current evidence gap of the service model configuration and associated costs to identify what is known and which gaps remain. It further develops research undertaken within a broader programme of work, that had sought to define the service models to identify their scope and scale ( Taylor *et al.*, 2023).

### Determining a typology of service models for intellectual disability and BtC

MELD (Mapping and Evaluating Services for Children with Learning Disabilities and Behaviours that Challenge) is a programme of research funded by the United Kingdom’s National Institute for Health Research (UK NIHR) to map English NHS community services for children with intellectual disability and BtC, to describe service models, and evaluate the outcomes and costs for these service models ( Taylor *et al.*, 2023). Researchers in the MELD collaborative team identified 278 potentially eligible services across England, and used complementary approaches to determine a typology of ‘service models’ that grouped similar community-based health and care services for children with intellectual disability and BtC. Services were excluded if they were a special school service, an inpatient service; or were not yet operational ( Taylor *et al.*, 2023). Latent Class Analysis was used with survey data gathered from 161 respondents to identify two service models. Descriptive analysis was then used to incorporate broader survey and interview data to inform final classifications, leading to the identification of five services models. Consultation with multiple advisory groups containing family carers and professionals (academic, practitioners) was undertaken to confirm the face validity of the service models’ typology ( Taylor *et al.*, 2023). Five service models were identified in this analysis. The distribution of all services varied across England; a fifth were in London (30/161~19%), similarly for the North-East and Yorkshire (29/161, ~18%) and the North-West (27/161~17%). Approximately fifteen percent were in the Midlands (23/161), the South-East (21/161) with around 10% in the South-West (17/161) and the East of England (14/161).

***Model 1: Child and Adolescent Mental Health Service (CAMHS) services (n= 69, 43%)***

Of the service models identified, the most common was a generic Child and Adolescent Mental Health Service (CAMHS) service. CAMHS broadly supports children with mental health needs, and usually offers a distinct intellectual disability treatment pathway to support children aged between 0 and 17. As part of the support framework, additional intensive supports for BtC may be offered.

***Model 2: ID CAMHS (n=28, 17%)***

Service model 2, ID CAMHS (Intellectual Disability CAMHS), is a service very similar to model 1, but is stand-alone and provides supports specifically for children with a diagnosed moderate-profound intellectual disability, or significant impairment of intellectual and social adaptive functioning that may include behavioural difficulties. ID CAMHS is identified as a separate model because it is not incorporated into a pathway within model 1 CAMHS.

The research identified that service models one and two together accounted for approximately sixty percent of services for children with intellectual disability.

***Model 3: Children/Young People’s Disability (n=25, 16%)***

Model three is a smaller service model focused on children with physical and other disabilities more generally, and includes supports for BtC in addition to other disability services. Nursing teams commonly provide support, including providing initial disability assessments.

***Model 4: Specialist BtC support (n=27, 17%)***

Specialist BtC services in model four represent approximately 17% of services and tend to be commissioned by local Integrated Care Boards, offering embedded intensive, or specialist supports such as Positive Behavioural Support (PBS) approaches. They include the use of private stand-alone children’s services prioritising BtC. Due to this model being less common across England than other models, and less common than adult service provision for BtC, families report that they struggle to identify or enrol their children in local specialist services. ( Hassiotis *et al.*, 2020; Hassiotis *et al.*, 2022).

***Model 5: all age (n=12, 7%)***

The smallest service model is quantified as being only a small number or approximately 7% of services, and offers an ‘all age’ (birth to adulthood, or from five years to adulthood) scope to the more generalised service, enabling a more seamless transition for children and young people into adult services. There may be a greater number of these service models available across England however, because adult services are reported to accept referrals for young people (age 14 to 17) in age transition ( Hassiotis *et al.*, 2020). These services tend to be geographically grouped in a similar region in England.

## Data collection methodology

Within each of the service models, sites delivering services for children with intellectual disabilities and BtC were identified in Study Survey One (Typology Mapping). The broader study received approval from the Humanities and Social Sciences Research Ethics Committee at the University of Warwick (REF: HSSREC 91/20-21. The sites were familiar with the research programme, they had already participated in the study’s typology mapping and held established research collaborative contracts with MELD. Informed written consent was obtained from all participants and participating organisations in the form of the signed research contracts. Following this, an initial survey (Stage One Survey) was sent to all sites requesting summary data in order to identify and map the service models. The research described in this manuscript was the second follow-up survey (Stage Two Survey) sent to the same sites, who had provided us with written consent in the form of the signed research collaborative agreements, in order for us to collect further detailed data about their staffing, service overheads, and cost structures.

Nineteen of the sites were considered for participation; five in model 1 CAMHS, three in model 2 ID CAMHS, three in model 3 Children/Young People’s Disability (CYP), five in model 4 PBS Behavioural Support and three in model 5 All Age. Sites were contacted by email by the MELD administrative team and invited to participate in the Costing Study. They were offered an online meeting with the health economics team for an introduction to the planned data collection scope, to include a fuller discussion of the BtC service at study onset. Initial and ongoing meetings with sites were held between June 2023 and June 2024.

Service level data was collected via a purpose-designed data collection instrument (survey). The survey was designed to be aligned to the format of the cost estimation templates used for national unit cost data reference sources, namely; the Compendia of the Unit Costs of Health and Social Care generated by the Personal Social Services Research Unit (PSSRU) and the University of York (CHE, York; Jones *et al.*, 2023). The objective of this approach was to document and to present the service data in a similarly disaggregate way. It was anticipated that one or more service leads would need to engage with the survey for completeness; likely a service manager, lead clinician and/or finance manager. The survey can be viewed in appendix 1. It was piloted with members of the MELD Study Management Group, and supplemented with interviews with specialist CAMHS Consultant Leads and subsequently revised with another two sites prior to circulation.

The survey was composed of seven sections. The introductory section presented customised summary data for each site, replicating each site’s data submission from the first mapping survey. The summary aimed to repeat identifiable features for each site, and to capture any changes (e.g. site mergers) that may have occurred in the interim. Data capture included the types of services offered, commissioning arrangements, site duration and permanence, inclusion criteria for intellectual disabilities and BtC, the age range for children and young people (CYP) with BtC and age of transition out of the service.

Section one explored the financial inputs for services for CYP with BtC. These included funding flows into the service, the use of private finance initiatives (PFI) for "public–private partnerships" (PPPs), charior philanthropic (not for profit) funding, for-profit funding or wrap-around services or other income generation activities. Cost-sharing across services was explored, for example between social services, education and health.

Sections two and three examined staffing as a key cost driver, including the staff team configuration by occupation, title and grade, the proportion of the role(s) funded for children with BtC, management and supervision responsibilities, duration of contact and ratio of direct to indirect time on client-related work. A narrative approach provided for free text that supported the data tables to accommodate for case-loads that varied, heterogeneity in clients’ individual characteristics, and their treatment needs.

Section four considered a conceptualised pathway of treatment following referral. It investigated the service reach to the eligible population, take-up of eligible users, mean time to assessment from the point of referral, mean length of time from commencement to discharge and finally, service gaps - such as waiting times.

Sections five and six investigated operational and overheads costs. They were intentionally positioned at the end of the survey so that the final section could be sent to the site’s finance manager for completion, as previous sections were usually completed by clinical leads or team managers. In this report, operational and overheads costs are reported at the beginning to provide a framework for all costs.

Section seven provided free text for miscellaneous information, to inform a comprehensive understanding of the variation in key service components, items or costs.

The gathered data were saved into a secure folder, and entered into a purpose-designed spreadsheet. Descriptive analysis was undertaken to explore and summarize the data, with tables developed for visualization. Common metrics include measures of central tendency (mean, median, mode) and measures of variability (range, variance, standard deviation) were estimated where appropriate, with missing data documented across all service model sites. Unstructured thematic analysis was used to explore the themes that emerged. When reporting the research findings, our survey data (survey 2) were triaged with the data from the stage 1 survey which had identified and mapped the service models, and had obtained a larger sample size and broader overarching summary statistics. This additional information provided context, and is referenced in the reporting of the research findings.

### Patient and Public Involvement

A MELD Parent Advisory Group (PAG) convened to provide lived-experience input during the analysis of this report. The group comprised parents and carers of children and young people with experience of mental-health and related support services. Engagement began during the proposal-development stage, when members of the Challenging Behaviour Foundation and the Parent Advisory Group helped refine the research focus on how community-based services for children with intellectual disabilities and behaviours that challenge (BtC) are structured and funded. Their insights, drawn from personal and professional experience, confirmed that understanding the organisation, costs, and accessibility of these services was a high priority for families but an under-researched area.

The Challenging Behaviour Foundation was a core partner in the project and met regularly with the research team to review progress, advise on emerging findings, and guide the framing of questions to ensure that the study reflected real-world family experiences and concerns. Members of the Parent Advisory Group were kept informed through regular updates and feedback sessions, they reviewed draft findings, and commented on the accuracy and sensitivity of interpretations.

While the Parent Advisory Group did not determine final conclusions, their perspectives shaped the framing of issues and the balance of priorities presented in this report. Their involvement helped ensure that the findings and outputs remained grounded in lived experience and relevant to the communities most affected.

## Results

For this research, of the nineteen sites contacted, ten sites returned some data. On average, a more complete set of data was returned by two sites in each service model; although more sites provided staffing data or additional data sections in an ad hoc manner. Some sites reported data for more than one locality in their jurisdiction. There was significant missingness in the data returns, both across and within data sections. A summary of data returns and data gaps is shown in appendix 3. The reporting approach undertaken here was inclusive, including all data returns to maximise comprehensiveness.

### Funding structure and funding flows into the sites

Table 1 below presents a summary of the funding flows and the proportion of children with BtC by service model.

**Table 1.** Summary of the funding flows and the proportion of children with BtC in the service models.

Model	Model 1	Model 2	Model 3	Model 4	Model 5
Category	CAMHS (n=2)	LD CAMHS (n=2)	C/YP disability (n=2)	Specialist BtC (n=2)	All age (n=1)
**Proportion of children with BtC**	One site an estimated sixty percent of case load is BtC	One site estimated all (100%) of their case load is BtC	Two sites defined by eligibility criteria Site one has a threshold for referral in – and no separate eligibility for BtC Site two, referrals are criteria led, triaged and individually assessed	One site notes their service is for both children and adults, fifty-four percent caseload are children, with forty three percent below age of fifteen.	Majority of referral include BtC Cost sharing adult-child for operational resourcing Cost sharing with Trust for estates and T&D
**Funding flows into the service**	Both sites annual block funding by the local Integrated Care Board One site receives additional funding from NHS England for Advanced Clinical Practitioner (ACP) trainees	One site is funded through an ICB, patients are referred in from a log of referrals	Block contract ICB Site refers out to other charities (no shared funding) No process for cost sharing/shifting	Funding from Local Authorities, or ICBs one site is a Limited Company one site is funded via Service Level Agreements	ICB support funding for staff accessed from a central fund in trust, monies accruing student and trainees on placement

***Model 1: CAMHS***

Data in the Stage One Survey was collected from seventy sites for model 1, with most sites reporting they were integrated into existing services such as CAMHS, with only five operating as stand-alone services. The Commissioning oversight for these services was primarily through a Clinical Commissioning Group (CCG) now incorporated into ICB, four by a Local Authority Social Care, two by NHS England Commissioners, and one is part of a Transforming Care Partnership. In this study, the two sites that returned data received block funding authorised by their local Integrated Care Board (ICB). One received additional funding from NHS England for their support of Advanced Clinical Practitioner (ACP) trainees. Neither made use of any private finance initiatives (PFI) for "public–private partnerships" (PPPs), for-profit funding, charitable or philanthropic funding, wrap-around services or additional income generating activities. One site estimated that approximately sixty percent of their case load was with children with BtC, with the other uncertain about their BtC caseload.

***Model 2: LD CAMHS***

The Stage One Survey identified that approximately seventy percent of sites function as stand-alone services. The remaining sites were integrated into other services. Of those, most were part of a broader Children and Young People's (CYP) service that includes CAMHS, while one is part of a National and Specialist Autism Spectrum Disorder (ASD)/Intellectual Disability (ID) service. The commissioning was predominantly through Clinical Commissioning Groups (CCGs), which oversee a total of 22 services. Three services were co-commissioned by a CCG, NHS England Specialist Commissioning, and a Transforming Care Partnership (TCP). One service was funded by an Integrated Care System (ICS) and another funded jointly by a CCG and Local Authority Social Care. Here, one site returned data and reported that they were commissioned by an ICB with all referrals into their service flagged as having challenging behaviours. The site did not make use of any private finance initiatives (PFI) for "public–private partnerships" (PPPs), for-profit funding, charitable or philanthropic funding, wrap-around services or additional income generating activities.

***Model 3: Children/Young People’s disability***

The majority of services were part of another service, while approximately thirty percent operated as stand-alone services. The integrated services were part of a diverse range of larger teams, including Children's Community Nursing, Community Learning Disability Teams, and Child Health Directorates. Several were integrated with Children and Young People's (CYP) services, and one was part of a Health Visiting service. Most were commissioned by Clinical Commissioning Groups and the Local Authority Social Care commissions nine services, with two also involving Local Authority Education. A few services had multiple commissioning sources, with one stand-alone service, for example, funded by a combination of NHS England, a Local Authority and a CCG, underscoring more complex funding arrangements.

Data returns for our survey identified block funding for sites authorised by the governing ICB. Their intellectual disability services were defined by eligibility criteria, with a threshold to be met for referral in, and with no separation for BtC. All referrals were criteria led, triaged and then individually assessed. CYP were referred onto other charities when appropriate, with no funding shared by services across or between sites, and no processes in place for cost sharing.

One site’s contract provided for a children’s community nursing service to all CYP who are registered with a GP and who meet the referral criteria, with specific treatment needs to then be individually assessed. The service was not split into workstreams hence no BtC-specific funding. Within the broader service (the Children’s Learning Disability Service) sits a team who support CYP with moderate to profound learning disability, and their families. The service may also refer to charitable organisations offering support to a child, young person or their family.

The other site advertised services for families on the local County Council website. A key eligibility criterion for a CYP is an IQ of less than 70. Thresholds for engagement and support are determined by the Signs of Safety tool, with individualised care plans subsequently developed.

***Model 4: Specialist BtC***

The Stage One Survey data showed the integration of specialist services within a broader care infrastructure. The majority of services functioned as part of another service, such as a sub-team or pathway, with services integrated into CAMHS teams, Community Children's Learning Disability teams, and Children's Social Care directorates. The majority of services were funded through CCGs being the most frequent funding body, and with services co-commissioned with Local Authority Social Care Local Authority Education. Transforming Care Partnerships (TCPs) and NHS England specialist commissioning underscores the collaborative and multi-agency nature of service provision.

Our data capture showed funding for two services provided via local authorities, with one service operating as a limited company through an ICB contract, and the other via service level agreements which generally provided for joint adult and child specifications and resource sharing, to account for fluctuating service demands. One site provided services for both adults and children, and with approximately fifty-four percent of the caseload being children. Of this, around forty-three percent of the service caters for children below the age of fifteen.

***Model 5: All age***

Most services were part of a larger team or service, with only one identified as stand-alone service. Many services were integrated within broader Learning Disability services. For example, one service was part of a wider all-age Learning Disability Service that includes both children and adult nursing teams. Some services were also described as providing additional support to community learning disability teams. The commissioning of these services is primarily done by Clinical Commissioning Groups (CCGs), although there are instances where NHS England specialist commissioning, Local Authority Social Care, and Integrated Care Systems (ICS) are involved, either alone or in combination with CCGs.

Data capture for our survey included one service that reported the majority of their referrals included BtC. The service supported two councils, each providing different services for the population, differentiated by the availability of other programs funded by the council or charities. Block funding was authorised by the local ICB. Cost-sharing within the intellectual disability service (particularly with the adult Community Team for People with Learning Disabilities (CTPLDs)) occurred for management, administration and logistical resources (e.g. psychometrics, kit, PPE). Cost-sharing in estates for training and development occurred across adult and child services. There was no separation for the needs of children who required specialist ID care, and the majority of these referrals were related to BtC. A central fund in the NHS Trust provided additional support funding for staff, with funds accrued from student and trainee placements.

### Operational and overheads costs

***Model 1 – CAMHS***

One site returned data and reported that their capital investment was not specific to the intellectual disability treatment pathway, and it was therefore not possible to disaggregate costs such as direct staff costs or indirect costs such as capital or revenue overheads. Costs that could be disaggregated included buildings and on-costs with no additional costs accrued because the site owned the building.

***Model 2 – LD CAMHS***

No sites reported any operation or overheads costs

***Model 3 - C/YP disability***

One site returned data, noting that they were unable to separate BtC costs or intellectual disability specific costs from Community Nursing expenditure apart from staffing, which had been reported separately.

***Model 4 - Specialist BtC***

Data was returned of varying completion by one site. On average, the direct operational costs for the sites was £1.2 million, with costs accruing to land and capital estimated between £200,000 and £250,000. Direct and indirect overheads were relatively low, estimated together at approximately £5,000.

***Model 5 - All age***

No sites reported data for overheads and operational costs.

### Staffing

This section provides an overview of the numbers and case-mix of staffing configuration by service model site.

***Model 1: CAMHS***

Both sites returned data. Site one CAMHS had psychiatry services with nursing and psychology support. The team comprised a Consultant Child and Adolescent Psychiatrist, a Nurse Consultant, a Learning Disability Nurse Band 7 ACP apprentice, two Band 7 Learning Disability Nurses, a Band 3 Health Care Assistant, a peer support worker, and a Psychologist. It had eight staff in total, of varying seniority and expertise. All roles were funded at 100% for children with BtC.

In comparison, the other CAMHS site comprised fifteen multidisciplinary staff. These included an Operational Lead who oversees a Team Manager and a Psychological Lead. A Clinical Psychologist provides consultation though is independent of this structure. The team manager oversees a clinical lead, five nurses, two support workers and an OT specialist lead. Three psychologists are overseen by the Psychology Lead. The clinical lead manages the speech and language therapist and the OT Specialist Lead manages two Occupational Therapists. Administrative support is provided by two medical secretaries and one administrator.

***Model 2: LD CAMHS***

One site returned data. Staffing is delivered through Clinical Psychology, Nursing, Psychiatry, Occupational Therapy, Speech and Language Therapy, Music Therapy, and additional Support Workers. In total fourteen staff were listed as being part of the multi-disciplinary teams which provided services across two councils.

***Model 3: Children/Young People’s disability***

Two sites returned data. In the first site, the team comprised a clinical lead, a deputy team lead, support workers, and community nurses’ posts which were currently vacant. All are funded for children with BtC, with approximately equal (50/50) ratios of direct to indirect time spent on client-related work.

The second site reported mostly nursing staff, comprising a Band 6 team lead, a Band 6 nurse (part-time), Band 5 learning disability nurse, nursing associate and trainee nurse associate. This team is staffed according to whole service need with no specific allocation for BtC. The team leader is responsible for ensuring accurate triaging, staff allocations and ongoing supervision which equates to approximately 30% of that role. A second Band 6 nurse rotates across the community and the trust vulnerabilities teams. Generally, Band 6 nurses manage all high priority cases, and Band 5 nurses may support a BtC referral under guidance. A Band 4 post is currently vacant and three trainee nurse associates supports the team. These may support the Band 6 or Band 5 nurses with a BtC referral and initial assessment or resource development.

***Model 4: Specialist BtC***

Three sites returned data. Twenty-seven multi-disciplinary staff were part of the team for one site and the bands are part of a local authority employment pay structure. Staff included a Band 11 Principal Manager, six Band 10 Practice Managers, four Band 9 Senior Behaviour Analysts, seven Band 8 Assistant Behaviour Analysts, two vacant posts for Band 8 Assistant Behaviour Analysts, two Band 3 Behavioural Practitioners, two vacant posts for Band 3 Behavioural Practitioners, an External Clinical Supervisor, a Clinical Psychologist and Administrative Support. Supervision of staffing approximated for forty percent of the Principal Manager’s role and the ratio of direct to indirect time on client-related work varied by staff. The Principal Manager had overall managerial responsibility for the service. The Practice Managers have a dual clinical and managerial role, and hold cases as behaviour analysts. They lead assessments, develop PBS plans and intervention, and oversee support provided. They also take responsibility for line management of staff and area management as part of the Service Level Agreements. There are four Senior Behaviour Analysts that have a purely clinical role, and hold cases as behaviour analysts. They will lead assessments, develop PBS plans and oversee the treatment delivery. The seven Assistant Behaviour Analysts assist the Senior Behaviour Analysts and Practice Managers to progress clinical work, whilst the Behaviour Practitioners provide direct support to caregivers to put support plans and intervention in to practice. The team has four vacancies (two Assistant Behaviour Analysts and two Behaviour Practitioners). More recently the team have been supported with administrative assistance.

The second site comprised a Consultant Psychiatrist, two Band 7 Nurse Specialists, Band 6 Community Practitioner, Band 4 Assistant Practitioner, Band 3 Assistant Practitioner, Occupational Therapist, eight Speech and Language Therapists, a Service Lead, a Board-Certified Behaviour Analyst, and a Trainee Behavioural Therapist. Approximately fifty percent of a Consultant Psychiatrist role is funded for children with BtC, with all other staff roles fully dedicated to BtC. Management activities and supervision of staffing differed by staffing and grade, primarily undertaken by the Band 7 Nurse Specialists and the Consultant Psychiatrist. Ratio of direct to indirect time on client-related work also varied, but tended to be split at around 50:50. Band three and four Assistant Practitioners spent the greatest amount of time with the children and young people with BtC, engaging in support of up to three visits weekly with between two and twenty-four hours of support per week. On average, the other staff spent approximately two hours with children and young people.

In the third site, a Service Lead sits over a team of Board-Certified Behavioural Analyst and Behaviour Technicians. All other staff roles were fully dedicated to BtC. The service lead spends approximately a quarter of their time in management and supervisory responsibilities, and is responsible for supervision, network engagement, contract management and in providing direct support to families and clients. The staff provide a day per week of client support, which includes family carers. The average time spent providing treatment support to any individual (and their support network) is approximately twenty-four weeks.

***Model 5: All age***

One site reported two psychologists (Band 8a and 8b) providing service cover of 1.8 FTE and occupational therapy (Band 7 and Band 6) equivalent to 1.4 FTE. Five nurses provided a total of 4.8 FTE and this team included a lead Band 7 nurse, two Band 6 learning disability nurses and two Band 5 learning disability nurses, with a bank nurse currently filling a vacancy. The team was also supported by a clinical assistant (Band 4) and administrative support. All staff roles were fully funded for children with BtC, though the clinical psychologists and the occupational therapists tended to spend more time engaged in supervision and management responsibilities, with nursing staff delivering more direct client related work. The team’s caseload at the time of data collection was 288 children and young people with BtC, and the total number of referrals to the children’s Community Team for People with a Learning Disability (CTPLD) in the previous twelve months was 262. On average, the time spent providing treatment support to any individual and their kinship-network is between three months to a year. This support could be longer in duration but the team are cognisant of ensuring equitable support for families on the waiting list.

A second site had an in-post psychiatrist, commissioned for a limited FTE per month and only for CYP with severe intellectual disabilities. A gap exists for children or young people with moderate intellectual disabilities whose treatment needs to be individually commissioned to receive psychiatric support. This is then provided by the psychiatry team in CAMHS, although CYP who are admitted to hospital as inpatients receive prompt psychiatric assessments. The site also had one clinical psychologist, senior and junior nurses, and a support worker. General Speech and Language Therapy services are external to the site and so families are referred on to other services and are typically added to a waitlist. Families also wait for a general paediatric occupational therapist to assess their home environment, though a current vacancy exists for specialist occupational therapist.

Table 2 presents the staffing complement by service model, as reported by sites.

**Table 2.** Summary of staffing complement by Service Model.

Model 1	Model 2	Model 3	Model 4	Model 5
Consultant child and adolescent psychiatrist, Nurse Consultant, LD Nurse band 6 X2, LD Nurse Band 7 ACP apprentice, Health Care Assistant Band 3, Peer support worker, Psychology ( *7 listed*) Operational lead. Psychological Lead. Consultation clinical Psychologist. Team manager. Clinical lead, 5 nurses, 2 support workers, an OT specialist lead and 3 psychologists. 2 medical secretaries and one Admin *(15 listed)*	Clinical Psychology, Nursing, Psychiatry OT, SLT, Music Therapy, Support Worker ( *14 listed*)	Team Lead and Clinician, Deputy Team Lead, Support Workers, Business Roles, Community Nurses (vacant) Band 6 Team Lead, Band 6 Vulnerabilities Rotational Nurse, Band 5 LD Nurse, Nursing Associate Trainee Nurse Associate	Band 11 Principal Manager, Band 10 Practice Manager (6), Band 9 Senior Behaviour Analysts (4), Band 8 Assistant Behaviour Analysts (7), Band 8 Assistant Behaviour Analysts (vacant) (2), Band 3 Behavioural Practitioners (2) Band 3 Behavioural Practitioners (vacant) (2), External Clinical Supervisor, Clinical Psychologist Administrative Support ( *27 listed*) Consultant Psychiatrist, Band 7 Nurse Specialist, Band 7 Nurse Specialist Band 6 Community Practitioner, Band 4 Assistant Practitioner, Band 3 Assistant Practitioner, Occupational Therapist, Speech and Language Therapist ( *8 listed)* Service Lead, BCBA, Trainee/ BT	Admin 1.0 2 psychologists- 1.8 5 nurses- 4.8 OT- 1.4 Clinical assistant- 1.0 ( *12 listed*)

Table 3 presents the data from sites per model in the form of a staffing case-mix for comparison. This report only shows reported roles and where possible, the numbers of staff per role. We did not obtain cost data for ranking, or thresholds for seniority and structure.

**Table 3.** Staffing case-mix.

	Model 1 CAMHS		Model 2 LD CAMHS		Model 3 C/YP Disability		Model 4 Specialist BtC		Model 5 All age	
	Site one	Site two	Site one	Site two	Site one	Site two	Site one	Site two	Site one	Site two
**Staffing (n)**										
Psychiatrist	1							1		
Clinical Psychologist		1	1	3	1		1		**1**	
Nurse Consultant	1	1		1						
Speech and Language Lead				1						
Occupational Therapy Specialist Lead		1		1	1			1		
Operational Lead										
Psychologist	1	1	1	1			1	3	2	
Occupational Therapist	1	1	2					1	2	
Speech and Language			2	1				1		
Senior Behavioural Analysts Band 9							4			
Behavioural Analysts Band 8							7			
Behavioural Analysts Band 7							2			
Nurse Band 7 (can also include ACP apprentice, LD nurse)	1	4		1				1	1	
Band 6 Nurse or Community Practitioner	1	7	6	2		1		1	6	
Band 5 Nurse					2	1				
Nursing Associate or Assistant Practitioner Band 4				1		1				
Music Therapist			1							
(Peer) Support worker		2	1		3					
Band 3 Health Care Worker or Behavioural Practitioner or Clinical Assistant						1		3		
Community Worker										
										
Team/ BusinessManager					2	1	1			
Practice Manager				1			5			
Medical Secretary	1	1								
Administrator							1		1	
										

The table above presents a matrix of the staff composition in the different service models. All teams were multi-disciplinary and with Band 6 and 7 nursing staff (including health care assistants or support workers) the most common. Sites in model one CAMHS and model four (Specialist BtC) provided Child and Adolescent Psychiatry. The other sites were led by a Consultant Clinical Psychologist, and clinical psychologists were present in all models. Model 3 (C/YP Disability) reported teams that primarily comprised nursing staff, and were smaller in scale, reporting less staff per site. Model 4 (Specialist BtC) utilised behavioural practitioners as core staff, complemented by a range of allied health professionals (occupational therapists, psychologists and speech and language therapists).

No sites with the exception of models one and four reported professional staff in leadership (e.g. Team Managers) and clinical leads seemed likely to perform combined clinical and managerial leadership and oversight. No sites provided data in response to queries about other contractual arrangements, recruitment incentives such as agency fees, or additional staffing commitments such as on-call contracting.

### Triage with Stage 1 Survey - Staffing data returns

The detailed data presented above is juxtaposed with a narrative of staffing data returns from the Stage One Survey.

***Model 1 – CAMHS***

Stage One Survey used a structured template for documenting numbers of staff. The number of Psychiatrists was varied, with one site having five, four sites having three, ten sites having two, and thirty-six sites having one. Clinical Psychologists and Assistant Psychologists were also represented across the distribution, with twenty-nine sites employing one, ten sites employing two, and the rest of the sites employing between three and seven. Assistant Psychologists ranged between one site employing five and twenty-two sites employed one. Learning Disability Nurses, Mental Health Nurses, Occupational Therapists, Qualified Teachers, Social Workers, Support Workers, and Speech and Language Therapists were all frequently represented. No sites reported physiotherapists, or dieticians. A diverse range of other roles were also flagged without title/salary grades or bands but that included Consultant Paediatricians, Specialist CAMHS Clinician, Art Therapist, Mental Health Practitioner, CAMHS practitioners, Creative Therapist, Psychotherapist, Peer Support Worker, Specialist Practitioners, Music Therapists, LD CAMHS Practitioner, Behaviour Therapist, Non-Medical Prescriber, Systemic Practitioner, Assistant Practitioner, and Behaviour Analyst. Further roles included Behaviour Analysts, Counselling Psychologist and Family Therapists, Health Visitor (HV), a Registered Sick Children's Nurse (RSCN), a Trainee High Intensity Therapist, and an Educational Mental Health Practitioner.

***Model 2: LD CAMHS***

Of the sites represented, a significant number employed Clinical Psychologists, with twenty sites having at least one. Clinical Psychologists ranged per site from one to five. Learning Disability Nurses are also widely employed, in nineteen sites. The number of Learning Disability Nurses ranged from one to eleven, with a median of three. Psychiatrists were employed in eighteen sites and Mental Health Nurses in eight with between one and two per site. Other professional roles were present but in fewer sites, Assistant Psychologists are employed in seven sites, with a range of one to four staff. Occupational Therapists were employed in eight sites, with a range of one to three staff. Speech and Language Therapists are present in five sites, ranging from one to two staff. Social Workers are present in five sites, with a range of one to four staff. General Nurses are present in three sites, with one to two staff. Health Care Assistants are reported in five sites. Other roles included Behaviour Therapists, Assistant Practitioners, and Psychotherapists. These roles are present in 19 sites, and the number of staff per site is between one and two. No sites reported employing an Assistant Social Worker, Dietician, or Physiotherapist.

***Model 3: Children/Young People’s Disability***

Support workers (averaging between 2–4 per site, and up to fourteen) were the highest filled role employed followed by Learning Disability Nurses (ranging between one and five per site, extending to eight or twelve). Clinical Psychologists and Social Workers were the next most frequently employed role. Other roles included Occupational Therapists, and Health Care Assistants. There was a lot of missing data in the returns for these roles and so it is difficult to accurately report staffing numbers by roles with confidence. Other noted roles included Business Support Worker, Emotional Health Worker, Specialist SEND Health Visitors, Education and Short Break Support Staff, Children's Nurses, Occupational Therapy Assistants; Residential Social Care Worker, Peer support, Portage Workers, SCIP workers (Supporting Change in Partnership), Short Breaks Reviewing Officer and Assistant Practitioner.

***Model 4: Specialist BtC***

Clinical Psychologists, Assistant Psychologists, Learning Disability Nurses and Support workers are highly prominent and were well-represented across multiple teams. Psychiatrists and Mental Health Nurses, Allied Health professionals such as Occupational Therapists and Speech and Language Therapists are also common. Roles unique and frequent in this service model included Behavioural Analysts and Practitioners supported by Assistant Behaviour Analysts and Behaviour Analyst Trainees. These roles are supported by Specialist worker, Intervention workers for Early Years, Residential Care Workers, Adolescent Support Services, Team Managers and Registered Nursing Associates.

***Model 5: All age***

The three roles represented in this service were Learning Disability Nurse (the most numerous), Clinical Psychologist and Support Workers and Health Care Assistants. Sites reported fractional employment of Psychiatrists and Allied Health Professionals (Speech and Language Therapist and Occupational Therapists). Other roles employed in the sites included Paediatric Nurses including nursery nurses.

A broad range of staff roles were documented across the five service models.

### Staffing as a cost driver

Staffing type, grade and configuration is a key cost driver in a BtC service. Appendix 2, Table A1. provides an overview of unit costs for the BtC staff reported in the service models. A few sites reported staff grades and salaries, but not all. This data therefore provides the national average annual cost and range of salaries attributable to all staff listed in Table 2. It presents resource use items, with an associated unit cost or range of unit costs (£), unit of analysis and the source of the unit cost including the reference page and table number. Unit costs were obtained from a variety of sources, to derive the most accurate estimates of cost data. Costs were extracted directly from published reports and were inflated to current prices, primarily from the Compendium of the Unit Costs of Health and Social Care 2023, Personal Social Services Resource Unit (PSSRU) ( Jones *et al.*, 2023).

Salary estimates obtained from national data for lead clinical and professional staff tend to be broadly similar - upwards of £100,000. Allied health professionals of an equivalent grade also tended to receive similar remuneration. Senior behavioural analysts’ salaries were at the top of the range for allied health professionals (£80,000 to £90,000), followed by behavioural analysts, psychologists and occupational therapists. Core nursing staff in all sites tended to be provided by Band 7 and Band 6 nurses, though a full spectrum of nursing support was provided by Bands 4 to 8. These salaries tend to range between £25,000 and £50,000. Once salary on-costs, qualifications and overheads were added to the benchmark salary, total estimates for staff costs increased by two or three-fold.

The true staff salary costs will vary enormously due to contractual agreements, recruitment incentives in particular agency fees, and unreported activities such as on-call contracting. There is considerable variability in the actual salaries paid to staff and in the monies accruing to sites due to flexible funding arrangements. There was also variation in the types of supervision provided by staff, and diversity in their activities. Some conduct home visits, school visits, work primarily from home or from an office, with teams and groups and might host inpatient and outpatient clinics. A psychologist may visit a school to observe a CYP there, and to support the teachers with strategies to help manage BtC. There was also substantial variability in the funding approaches to staff activity, and some staff undertook significant travel. Training was highly variable between trusts and roles, and most sites did not report on their consumables.

Sites may not have provided comparable data, making interpretation difficult. Broader staffing costs, such as those for on-call, management, and administration, are highly variable depending on how they were declared in the survey. It is often complicated and difficult to separate out costs for shared management, administration, and building-related expenses, particularly if these functions were part of a larger, higher-level service architecture and were not fully included in the estimates.

It is also important to consider whether staff roles were missed that were accessed through shared services across the wider organization. For instance, while a service might not directly employ a psychiatrist or an occupational therapist in a dedicated role, they could still access the professionals from parallel teams. Many survey responses were incomplete, and the raw staff numbers presented are likely insufficient and could be misinterpreted; the staffing cost data should therefore be viewed cautiously due to significant missing information. To provide a more accurate measure of the total workforce, both staff roles that are dedicated and accessed through shared services should be described in full-time equivalence (FTE).

### Reported staffing organogram and associated costs

Exploring staffing configuration and cost data across services models is known as benchmarking. It aims to provide granular insight into a service’s largest resource and usually greatest expense, thereby enabling strategic, data-driven caparisons in terms of staffing complement. When comparing similar health and social care services (within model comparisons), decision-makers are able to identify how their resources are allocated compared to their peers to achieve similar objectives. This can reveal differences in operational approaches to staffing activities, procurement or service delivery. Through documenting and sharing data on performance metrics like staff costs, service wait times, or quality of outcomes, decision-makers can strategically review services and identify opportunities for continuous improvement. By the same token, staffing differences across service models highlight the resources available within the broader complex health and care ecosystem.

The following tables attempted to present actual costings attributable to staff in the different service models, and to represent data returns. Costings reflect actual reported grade and salary data where possible, with estimates used from national averages where the data was missing. Due to the lack of accuracy of apportionment for overheads and operating costs, a pragmatic approach to apportion overheads to staffing was undertaken, estimated from the calculations provided in the Compendium of the Unit Costs of Health and Social Care.

***Model 1 – CAMHS***

**Site one**

**Table:** 

staff role	funding type	pay grade	Salary (£)	%FTE	Total £ (WTE) adjusted *including salary, on costs, qualifications, management and capital overheads, and travel.	Total £ adjusted for %FTE
Consultant Psychiatrist	NHS	Consultant	159,062	0.4	363,727	145,490
Nurse Consultant	NHS	8b	75,535	0.6	171,208	102,725
Clinical Psychologist	NHS	8b	64,783	0.6	154,816	92,889
Advanced Nurse Practitioner	NHS	7	43,742	1	100,669	100,669
Nurse Practitioner	NHS	6	42,618	1	97,355	97,355
Occupational Therapist	NHS	6	35,392	1	87,976	87,976
Medical Secretary	NHS	2	47,941	0.21	181,742	38,165
**Total**					**1,157,496**	**665,273**

* Unit costs of health and social care calculator: Section 11.3.1 Unit cost component multipliers of hospital doctors, nurses and scientific staff Table 12.4.2 (doctors), Table 12.4.1 (allied health professionals), Table 13.x.x (hospital-based nurses) Total adjusted by baseline salary.

Site two **

**Table:** 

staff role	funding type	pay grade	Salary (£)	%FTE	Total £ (WTE) adjusted *including salary, on costs, qualifications, management and capital overheads, and travel.	Total £ adjusted for %FTE
Nurse Consultant	NHS	8b	56,907	Not known	128,986	
Clinical Psychologist	NHS	8a	49,149	Not known	118,326	
Clinical Psychologist	NHS	8a	49,149	Not known	118,326	
Nurse	NHS	7	42,376	Not known	97, 526	
Nurse	NHS	7	42,376	Not known	97, 526	
Nurse	NHS	7	42,376	Not known	97, 526	
Nurse	NHS	7	42,376	Not known	97, 526	
Nurse Practitioner	NHS	6	35,118	Not known	80,223	
Nurse Practitioner	NHS	6	35,118	Not known	80,223	
Nurse Practitioner	NHS	6	35,118	Not known	80,223	
Nurse Practitioner	NHS	6	35,118	Not known	80,223	
Nurse Practitioner	NHS	6	35,118	Not known	80,223	
Nurse Practitioner	NHS	6	35,118	Not known	80,223	
Nurse Practitioner	NHS	6	35,118	Not known	80,223	
Nurse Practitioner	NHS	6	35,118	Not known	80,223	
Occupational Therapist	NHS	6	35,118	Not known	80,223	
Assistant	NHS	3	28,074	Not known	64,493	
Assistant	NHS	3	28,074	Not known	64,493	
Medical Secretary	NHS	2	22,246	Not known	50,848	
**Total**					**1,267,479**	

* Unit costs of health and social care calculator: Section 11.3.1 Unit cost component multipliers of hospital doctors, nurses and scientific staff. Table 12.4.2 (doctors), Table 12.4.1 (allied health professionals), Table 13.x.x (hospital-based nurses). Total adjusted by baseline salary. ** Salary bands provided by site, costs taken from the Compendium of the Unit Costs of Health and Social Care

Both sites employ a Nurse Consultant, Clinical Psychologist, Nurse Practitioner, Occupational Therapist, and Medical Secretary. Site One employs a Consultant Psychiatrist and an Advanced Nurse Practitioner, roles not present at Site Two. Site Two employs general Nurses (7) and Assistants (3), which are not listed at Site One. The tables provide FTE and FTE-adjusted-costs for Site One, but this data is not available for Site Two. Therefore, a direct comparison of total FTE adjusted costs between the two sites is not possible.

***Model 2 – LD CAMHS***

Site one **

**Table:** 

staff role	funding type	pay grade	Salary (£)	%FTE	Total £ (WTE) adjusted *including salary, on costs, qualifications, management and capital overheads, and travel.	Total £ adjusted for %FTE
Clinical Psychology Lead	NHS	8b	104,450	0.56	244,082	136,685
Clinical Psychologist	NHS	8a	49,149	1	118,326	118,326
Nurse	NHS	6	35,118	1	80,223	80,223
Nurse	NHS	6	35,118	0.75	80,223	60,167
Nurse	NHS	6	35,118	0.75	80,223	60,167
Nurse	NHS	6	35,118	0.75	80,223	60,167
Nurse	NHS	6	35,118	0.56	80,223	44,924
Nurse	NHS	6	35,118	0.56	80,223	44,924
Occupational Therapist	NHS	6	34,736	0.75	86,346	64,759
Occupational Therapist	NHS	6	34,736	0.45	86,346	38,855
Speech and Language Therapist	NHS	6	34,736	0.56	86,346	48,353
Speech and Language Therapist	NHS	6	34,736	0.56	86,346	48,353
Support Worker	NHS	3	23,387	1	59,886	59,886
**Total**					**1,249,016**	**865,789**

* Unit costs of health and social care calculator: Section 11.3.1 Unit cost component multipliers of hospital doctors, nurses and scientific staff Table 12.4.2 (doctors), Table 12.4.1 (allied health professionals), Table 13.x.x (hospital-based nurses) Total adjusted by baseline salary. ** Salary bands provided by site, costs taken from the Compendium of the Unit Costs of Health and Social Care

Site two **

**Table:** 

staff role	funding type	pay grade	Salary (£)	%FTE	Total £ (WTE) adjusted *including salary, on costs, qualifications, management and capital overheads, and travel.	Total £ adjusted for %FTE
Specialist Clinical Psychologist	NHS	8a	100,586	0.8	235,053	188,042
Specialist Clinical Psychologist	NHS	8a	100,586	0.6	235,053	141,031
Specialist Clinical Psychologist	NHS	8a	100,586	0.5	235,053	117,526
Clinical Lead/Speech and Language Therapist	NHS	8a	104,450	1	244,082	244,082
Child and Educational Psychologist	NHS	7	42,376	0.5	97,526	48,763
Highly Specialist LD Nurse / Trainee Nurse Prescriber	NHS	7	43,742	0.6	100,669	60,401
Specialist Children's Occupational Therapist	NHS	6	35,118	1	80,223	80,223
Specialist Children’s Speech & Language Therapist	NHS	6	35,118	0.75	80,223	60,167
Psychological Therapies Practitioner	NHS	5	28,074	0.75	64,439	48,329
Psychological Therapies Practitioner	NHS	5	28,074	0.75	64,439	48,329
Specialist Children’s Occupational Therapist	NHS	5	28,074	0.56	64,439	36,085
Clinical Associate Psychologist	NHS	5	28,074	0.56	64,439	36,085
Clinical Coordinator	NHS	4	22,246	1	50,848	50,848
**Total**					**1,616,486**	**1,159,911**

* Unit costs of health and social care calculator: Section 11.3.1 Unit cost component multipliers of hospital doctors, nurses and scientific staff Table 12.4.2 (doctors), Table 12.4.1 (allied health professionals), Table 13.x.x (hospital-based nurses) Total adjusted by baseline salary. ** Salary bands provided by site, costs taken from the Compendium of the Unit Costs of Health and Social Care

The two sites have distinct differences in their workforce structure and costing profiles. While they both employ a Clinical Psychologist, Nurses, Occupational Therapists, and a Speech and Language Therapist. Site Two's staff appears to have more specialization and senior roles. It has three Specialist Clinical Psychologists and a Clinical Lead/Speech and Language Therapist, whereas Site One has a single Clinical Psychology Lead. Site Two also includes a Clinical Coordinator and Clinical Associate Psychologist, roles not present at Site One. In terms of staffing mix: Site One employs a Support Worker, while Site Two employs Psychological Therapies Practitioners and a Highly Specialist LD Nurse. Site One has more reported full-time roles (2.5 FTEs), while Site Two has only one full-time role (1 FTE). Site One's staff seems to be primarily part-time, with varying FTE percentages. Site Two's total adjusted cost is higher than Site One's, reflecting the higher overall cost of its workforce even when adjusted for part-time work.

***Model 3 - C/YP disability***

Site one **

**Table:** 

staff role	funding type	pay grade	Salary (£)	%FTE	Total £ (WTE) adjusted *including salary, on costs, qualifications, management and capital overheads, and travel.	Total £ adjusted for %FTE
Team Lead and Clinician	ICB	Not recorded	100,586	0.94	235,053	220,949
Deputy Team Lead	ICB	Not recorded	82,843	1	195,078	195,078
Support Worker	ICB	Not recorded	23,387	1	59,886	59,886
Support Worker	ICB	Not recorded	23,387	0.56	59,886	33,536
Support Worker	ICB	Not recorded	23,387	0.56	59,886	33,536
Business role	ICB	Not recorded	47,941	0.5	181,742	90,871
Business role	ICB	Not recorded	47,941	0.5	181,742	90,871
Community Nurse	ICB	Not recorded	35,118	1	80,223	80,223
Community Nurse	ICB	Not recorded	35,118	0.6	80,223	48,133
**Total**					**1,133,719**	**853,083**

* Unit costs of health and social care calculator: Section 11.3.1 Unit cost component multipliers of hospital doctors, nurses and scientific staff Table 12.4.2 (doctors), Table 12.4.1 (allied health professionals), Table 13.x.x (hospital-based nurses) Total adjusted by baseline salary. ** Salary bands provided by site, costs taken from the Compendium of the Unit Costs of Health and Social Care

Site two **

**Table:** 

staff role	funding type	pay grade	Salary (£)	%FTE	Total £ (WTE) adjusted *including salary, on costs, qualifications, management and capital overheads, and travel.	Total £ adjusted for %FTE
Team Lead	ICB	6	75,535	Not recorded	171,208	
Vulnerabilities Rotational Nurse	ICB	6	43,742	Not recorded	100,669	
LD Nurse	ICB	5	35,118	Not recorded	80,223	
Nursing Associate	ICB	4	28,074	Not recorded	64,439	
Trainee Nurse Associate	ICB	2	22,246	Not recorded	50,848	
**Total**					**467,387**	

* Unit costs of health and social care calculator: Section 11.3.1 Unit cost component multipliers of hospital doctors, nurses and scientific staff Table 12.4.2 (doctors), Table 12.4.1 (allied health professionals), Table 13.x.x (hospital-based nurses) Total adjusted by baseline salary. ** Salary bands provided by site, costs taken from the Compendium of the Unit Costs of Health and Social Care

Both sites are funded by the Integrated Care Board (ICB), and employ a Team Lead and at least one type of Nurse. Site One has a larger workforce with 9 staff members, and a wider variety of roles, including Deputy Team Lead, Support Workers, and Business roles, which are not present at Site Two. Site Two reports a workforce of five staff, and a more focused nursing team, with specific roles like Vulnerabilities Rotational Nurse, LD Nurse, Nursing Associate, and Trainee Nurse Associate. Site One provides detailed FTE percentages for each staff member. This information is not recorded Site Two, making it impossible to calculate adjusted costs based on hours worked.

***Model 4 - Specialist BtC***

**Site one – service 1**

**Table:** 

staff role	funding type	pay grade	Salary (£)	%FTE	Total £ (WTE) adjusted *including salary, on costs, qualifications, management and capital overheads, and travel.	Total £ adjusted for %FTE
Service Lead	Employee	Not stated	40,000–45,000	1	100,669	
BCBA	Employee	Not stated	32000–39,000	1	86,346	
Trainee/ BT	Employee	Not stated	27,000–32,000	1	64,439	
**Total**					**251,454**	

* Unit costs of health and social care calculator: Section 11.3.1 Unit cost component multipliers of hospital doctors, nurses and scientific staff Table 12.4.2 (doctors), Table 12.4.1 (allied health professionals), Table 13.x.x (hospital-based nurses) Total adjusted by baseline salary.

Site two **

**Table:** 

staff role	funding type	pay grade	Salary (£)	Mid-point (£)	%FTE	Total £ (WTE) adjusted *including salary, on costs, qualifications, management and capital overheads, and travel.	Total FTE N/A
Principal Manager	Not recorded	11	48,474– 51,515	49,994	Not recorded	118326	
Practice Manager	Not recorded	10	44,428– 47,420	45,924	Not recorded	100,669	
Practice Manager	Not recorded	10	44,428– 47,420	45,924	Not recorded	100,669	
Practice Manager	Not recorded	10	44,428– 47,420	45,924	Not recorded	100,669	
Practice Manager	Not recorded	10	44,428– 47,420	45,924	Not recorded	100,669	
Practice Manager	Not recorded	10	44,428– 47,420	45,924	Not recorded	100,669	
Senior Behaviour Analyst	Not recorded	9	40,221– 43,421	41,821	Not recorded	97, 526	
Senior Behaviour Analyst	Not recorded	9	40,221– 43,421	41,821	Not recorded	97, 526	
Senior Behaviour Analyst	Not recorded	9	40,221– 43,421	41,821	Not recorded	97, 526	
Senior Behaviour Analyst	Not recorded	9	40,221– 43,421	41,821	Not recorded	97, 526	
Assistant Behaviour Analyst	Not recorded	8	36,648– 39,186	37,917	Not recorded	86,346	
Assistant Behaviour Analyst	Not recorded	8	36,648– 39,186	37,917	Not recorded	86,346	
Assistant Behaviour Analyst	Not recorded	8	36,648– 39,186	37,917	Not recorded	86,346	
Assistant Behaviour Analyst	Not recorded	8	36,648– 39,186	37,917	Not recorded	86,346	
Assistant Behaviour Analyst	Not recorded	8	36,648– 39,186	37,917	Not recorded	86,346	
Assistant Behaviour Analyst	Not recorded	8	36,648– 39,186	37,917	Not recorded	86,346	
Assistant Behaviour Analyst	Not recorded	8	36,648– 39,186	37,917	Not recorded	86,346	
Behaviour Practitioner	Not recorded	3	23,500– 23,893	23,696	Not recorded	59,886	
Behaviour Practitioner	Not recorded	3	23,500– 23,893	23,696	Not recorded	59,886	
External clinical supervisor BCBA- D, Clinical Psychologist	Not recorded		100,586	100,586	Not recorded, assumed 0.5% FTE	235,053	
Admin support	Not recorded		22,246		Not recorded	50,848	
**Total**						**1,631766**	

* Unit costs of health and social care calculator: Section 11.3.1 Unit cost component multipliers of hospital doctors, nurses and scientific staff Table 12.4.2 (doctors), Table 12.4.1 (allied health professionals), Table 13.x.x (hospital-based nurses) Total adjusted by baseline salary. ** Salary bands provided by site, costs taken from the Compendium of the Unit Costs of Health and Social Care

Site three**

**Table:** 

staff role	funding type	pay grade	Salary (£)	%FTE	Total £ (WTE) adjusted *including salary, on costs, qualifications, management and capital overheads, and travel.	Total £ adjusted for %FTE
Consultant Psychiatrist	NHS	7	57,702	1	118,326	118,326
Nurse Specialist	NHS	7	57,702	1	100,669	100,669
Qualified Nurse	NHS	7	57,702	1	100,669	100,669
Community Practitioner	NHS	6	46,619	1	97,526	97,526
Assistant Practitioner	NHS	4	32,547	1	86,346	86,346
Assistant Practitioner	NHS	4	32,547	1	86,346	86,346
Occupational Therapist	NHS	6	46,619	1	97,526	97,526
Speech and Language Therapist	NHS	6	46,619	1	97,526	97,526
Consultant Psychologist	NHS	8c	76,035	0.6	163,098	97,858
Assistant Psychologist	NHS	5	57,525	1	65,559	65,559
Assistant Psychologist	NHS	5	57,525	1	65,559	65,559
Psychologist	NHS	8a	40,893	0.6	118326	70,995
**Total**					**1,197,476**	**1,084,905**

* Unit costs of health and social care calculator: Section 11.3.1 Unit cost component multipliers of hospital doctors, nurses and scientific staff Table 12.4.2 (doctors), Table 12.4.1 (allied health professionals), Table 13.x.x (hospital-based nurses)

All three sites have significant differences in their staffing and costs, with Site Two having the most complex structure. All three sites have a leadership role (Service Lead, Team Lead, Consultant Psychiatrist), however there are no roles that are identical across all three sites. Site Two has the largest team with 21 staff members, followed by Site Three with 12, and then Site One. Site One has a small, specialized team focused on behavioural analysis, including a BCBA (Board Certified Behaviour Analyst) and a Trainee BT (Trainee Behavioural Technician). Site Two has a large hierarchy of management and behavioural analysis roles, including multiple Practice Managers, Senior Behaviour Analysts, and Assistant Behaviour Analysts. It also includes Behaviour Practitioners and an Admin Support role. Site Three has a more traditional clinical team, with a Consultant Psychiatrist, various Nurses, and different types of Psychologists and Therapists. Site One and Site Three have full-time staff, but Site Three also includes multiple part-time positions. The FTE data for Site Two is not recorded. A direct comparison of individual FTE costs is difficult due to the differing staff roles and salaries.

***Model 5 - All age***

Site one **

**Table:** 

staff role	funding type	pay grade	Salary (£)	%FTE	Total £ (WTE) adjusted *including salary, on costs, qualifications, management and capital overheads, and travel.	Total £ adjusted for %FTE
Principal Clinical Psychologist	NHS	8b	104,450	0.8	244,082	195,265
Senior LD Nurse	NHS	7	44,973	1	100,669	100,669
Senior Occupational Therapist	Not stated	7	58,212	0.4	103,935	41,574
Occupational Therapist	Not stated	6	34,736	1	86,346	86,346
LD Nurse	Not stated	6	37,577	1	80,223	80,223
LD Nurse	Not stated	6	37,577	1	80,223	80,223
LD Nurse	Not stated	6	37,577	0.6	80,223	48,133
LD Nurse	Not stated	5	30,241	1	64,439	64,439
LD Nurse	Not stated	5	30,241	1	64,439	64,439
LD Nurse	Not stated	5	30,241	0.8	64,439	51,551
**Total**					**969,018**	**812,862**

* Unit costs of health and social care calculator: Section 11.3.1 Unit cost component multipliers of hospital doctors, nurses and scientific staff Table 12.4.2 (doctors), Table 12.4.1 (allied health professionals), Table 13.x.x (hospital-based nurses) ** Salary bands provided by site, costs taken from the Compendium of the Unit Costs of Health and Social Care

Only one site in model 5 reported staffing data.

There is considerable uncertainty in the costs presented in the data tables above due to ad hoc data completion and data missingness. Actual salary data provided by sites tended to be higher than costs estimated for the same band from the Unit Cost Compendium. Total costs were adjusted to include salary, on costs, qualifications, management and capital overheads, and travel and these were derived from the Compendium, based on either the original recorded salary or the reported grade.

Benchmarking supports decision-makers to determine whether the current distribution of resources (e.g. staffing) across the broader service model is optimal. A valuable review will extend an appraisal from "how much does this service cost?" to "how is this service model structured to achieve its objectives, and how can it become more effective?" A comparison of the five models reveals both similarities and differences in their staffing and cost structures. There is a presence of specific clinical roles across all models and nurses, psychologists, and therapists (occupational or speech and language) which are found in at least three of the five models.

- Model 1: CAMHS has a varied, generalist clinical team, including Consultant Psychiatrists, Nurse Consultant, and Advanced Nurse Practitioner and a large range of diverse support roles.
- Model 2: LD CAMHS has a strong focus on specialist roles, with multiple Specialist Clinical Psychologists, a Clinical Lead, and a LD Nurse.
- Model 3: C/YP Disability has a split funding source and a simpler structure. Site One focuses on general clinical and support roles, while Site Two is heavily concentrated on nursing roles at varying experience levels.
- Model 4: Specialist BtC has a unique focus on behavioural analysis, with roles like a BCBA, Behaviour Analyst, and Behaviour Practitioner with support from Support Workers.
- Model 5: All Age is dedicated to ID with a team primarily comprised of various ID Nurses and an Occupational Therapist and paediatric nursing and support staff.

The models vary in size and cost. The large difference in total costs and staff numbers highlights different operational scales and service delivery models.

Whilst significant data were missing for staffing costs for this section of the report, narrative data were provided by sites for service configuration and reach to the community.

### Service configuration and reach to the community

***Model 1: CAMHS***

The two CAMHS sites differed in approach to their frequency of contact with families and associated activities, with the first site reporting a varied and somewhat unstructured approach.

The second site described a regular pathway with visits averaging approximately an hour every three weeks, and an intensive support pathway of weekly visits. On average, families were enrolled for a year, though the longest enrolment period was recorded as four years. Families or young people were invited to complete an initial assessment within the 28 days, and commencement is determined by internal wait lists which differs by specialty. At the time of data collection, the waitlist delay for nursing was two weeks, occupational therapy sixteen weeks, speech and language therapy seven weeks and psychology five weeks. Almost all eligible families engaged with the service when offered consultations. Eighty-three young people were open on the site’s caseload.

Stage One Survey data provided additional contextual information for Model 1. On the average the current total active caseload averaged 120 CYP with BtC, with a large range (minimum of 7CYP and a maximum of 835 CYP). Forty percent of sites had an active caseload of between one and a hundred CYP with BtC, and another ten sites had between a hundred and two hundred CYP with BtC.

One site reported almost 4000 referrals (new and re-referrals in), another 2750 and a third 1250. Three sites had referrals in of between five hundred and seven hundred CYP, and another three of between 250 and 400. Approximately fifteen sites received between 100 and 200 referrals annually, and thirty-five of up to a hundred a year.

***Model 2: LD CAMHS***

Staff allocated approximately eighty-percent of their working week to direct clinical activity. The mean time to starting treatment from referral varied by specialty (between one week and three months), but on the average would commence within three months. The average duration of contact is approximately fifteen months.

In site two, commencement of treatment from referral averaged between six and twelve months, but can vary due to clinical need and presentation, including the risks to the children due to their moderate to severe intellectual disability and BtC. Service uptake is high, with at least ninety percent of eligible users commencing treatment when offered. A waiting list is reviewed on a weekly basis, and waiting times can be reduced with escalations in treatment if necessary. There are no additional delays between diagnosis and treatment. The time frames for service delivery are variable and may be non-ending if psychiatry prescribe medications as part of the CYP’s treatment regimens. These will then be extended due to ongoing monitoring.

Feedback for the Stage One Survey for model 2 showed a quarter of sites are designed exclusively for CYP with BtC. The majority report that their services are not exclusively for this group, indicating that they manage a broader caseload that include individuals with other or co-occurring needs. Sites specified that they catered to children and young people (CYP) with a learning disability and moderate to severe mental health difficulties. These included a wide spectrum of presentations such as anxiety, depression, low mood, emotional regulation issues, self-harm, and sleep problems, ADHD, autism spectrum disorder (ASD) assessments, OCD-type behaviours, eating/feeding issues, and toileting/continence problems.

Similarly, the active caseloads show a broad distribution. Several services maintained caseloads of between two hundred and fifty to three hundred CYP. The lowest reported active caseload was eighteen, while the highest is 440. Most caseloads were between sixty and a hundred and sixty CYP.

The number of annual referrals (new and re-referrals) received by the services varied. Referrals in a typical year varied between twenty and up to 1300, highlighting a large disparity in service demand and capacity. Other services reported numbers of around two hundred, with many in between sixty to a hundred and fifty referrals in per year.

***Model 3: Children/Young People’s Disability***

Site three has a clinic-based network across a fairly large area covering a number of local authorities with variability in practice and approach. A key eligibility criterion for a CYP is an IQ of less than 70. Thresholds for engagement and support were determined by the Signs of Safety tool, with individualised care plans subsequently developed. However, the average time to starting treatment following referral is within 18 weeks’ (4–5 months), and uptake is generally good. Children and young people are triaged at initial referral, and are allocated high, medium, or low priority. BtC is usually allocated as high priority. The average number of sessions per child is between eight and fourteen. Staff are up to date with the waitlist as all caseloads are reviewed monthly for acuity and safety, and its therefore unlikely that a child may be in the waitlist system for long periods of time. A BtC treatment approach may include strategies and resources, and discharge may be offered in as little as six weeks with family, carer and education team engagement and support. Key support themes include toileting, sleep, fussy eating, behaviour, challenging behaviour, puberty and self-care. Discharge does not take place until all strategies are implemented and consistently maintained across CYP’s usual environment. Parents and carers of a CYP can be referred to a face-to-face or virtual workshop with other families, to include behaviour management techniques. Escalation for additional family support might include social care services, short break support with local services, additional health need support via children’s continuing care services and mental health or psychology support in CAMHS. There are generally no long-term patients of beyond twelve months in the caseload. Re-referrals are accepted after a six-month period for further review. The team offers telephonic support to families, and/or a new referral can be opened. Pressure on the service is immense with concerns that specific vulnerable cohorts are not receiving sufficient care. These include the age 16+ cohort, families where English is not the first language and families with learning disabled parents.

A more structured summary of the treatment pathway is detailed here. Patients are referred into the service, where they are offered functional behaviour and specialist sensory assessments; relationship and sex education (RSE), emotional and well-being work, and sleep assessments. Home and school observations are conducted. The referrals are screened, and thereafter evaluated for suitability. They are then placed on a waiting list.

Staff review initial assessments following family visits and decide on the treatment supports. One or multiple treatment pathways are offered to the patient depending on their need, although there are two primary pathways. The first is a clinic-based pathway in which children undertake play-based therapies or sensory assessments; the second is ‘out of clinic’ which is usually undertaken at school. Both treatments are delivered across five weeks. Family-based outreach is additionally delivered, supporting families through their implementation of treatment plans. This is usually conducted across several sessions, thereafter the case will be closed.

Data from the Stage One Survey show there is variation in the number of CYP with BtC on waiting lists and as active caseloads across the services. The active caseload data, shows almost a third of the services manage up to fifty cases, with another third managing up to a hundred cases. The remaining services manage higher caseloads, with a few having over two hundred active cases, including one with a caseload of over 1000.

The referral-in range is similarly broad, spanning a low of twenty-three to 5,200 referrals (an extreme outlier). A more representative measure is the median, which is approximately one hundred and fifty referrals per year. This significant variance in demand across the services may highlight a potential imbalance in capacity and need within different regions. For waiting lists, a few services reported no one on their waiting list, while others reported up to twenty-five. A small minority have up to fifty CYP people waiting, and only two services reporting more than fifty.

***Model 4: Specialist BtC***

In one site, the levels of consistent engagement can vary but it is rare for someone not to take up the treatment, and waiting times are high. Caregivers of the CYP placed on the waiting list are offered a training course. The training course is an introduction to positive behaviour support and basic training in the functional understanding of challenging behaviour. In some locations (depending upon local arrangements), caregivers may be directed to other services for support.

In the second site, initial consultations and subsequent treatment referral takes less than six weeks. As waiting times are high, care givers may be directed to other services for support, or provided with online training. Service commissioners attempt to ensure that service capacity is available to meet the demand, and referral pathways are managed through a dynamic support register (DSR). Eligible referrals are placed on the DSR; with the multidisciplinary team meeting monthly to review all cases. Approximately seventy young people and children are supported through the service annually, and at least eighty-percent of families who are offered treatment will engage. The average treatment duration is approximately six months, and other services in the borough may be signposted if considered to be more appropriate. The team may make initial assessments and then provide referral and recommendations to a treatment network outside of their own services.

Stage One Survey data showed that current active cases for CYP with BtC ranged between three and seventy cases per site, with a median of eleven. Most (ten sites) sites had no-one on the waitlist, and the few sites that did ranged from three to twenty-seven CYP with BtC. The referrals-in ranged from a minimum of four referrals to a maximum of two hundred and fifty referrals per annum. The average number of referrals is approximately thirty-eight (a median of 30).

***Model 5: All age***

The first site reported that their total referrals in the previous year were 262 and their current client caseload was 288. The average time from referral to assessment was approximately forty-five days with the DNA rate being very low. The average treatment duration is three months to a year, with families usually discharged within sixteen months because the team are cognisant of providing equitable services for families on the wait list. As part of the waitlist process, letters are sent to families to offer a speedier escalation and triage in the case of deterioration. The level of demand for the service is proving challenging and the team note they are constantly reviewing their resources to assess capacity and efficiencies. Unmet need is sometimes filled by trainee clinical psychologists and/or substitute offers of preventative short-term PBS-focused support.

The second site operates an open referral system, so anyone can be referred or can self-refer. Most referrals accrue from local schools and paediatricians. The CYP must meet the criteria for intellectual disability to be eligible for treatment. Patients are screened within twenty-four hours following referral to identify immediate risks, and assessments are conducted by paediatricians, with staff additionally engaging with schools. The team hold weekly review meetings, overseen by a clinical lead, nurses and psychologists. The families are then placed on the wait list, which is very long. Triage appointments are offered to families, with a nurse reviewing the treatment pathway. The treatment pathways that are offered differ, with behavioural interventions offered on the behaviour pathway delivered in line with NICE guidance. The service offers sensory and psychological support, and a sleep pathway delivered via a sleep clinic. Parent group workshops are recommended for lower level support for example providing information on emotional well-being, or through a teenage parenting programme called ‘Riding the Rapids’.

The Stage One Survey data noted that the current active cases for CYP with BtC ranged between zero and two hundred and ten cases per site, with a median of twenty-three. Data for the waitlist was missing. The referrals-in ranged from a minimum of two referrals per annum to a maximum of five hundred and fifty. The average number of referrals was approximately two hundred and forty (a median of 165).

## Discussion – strengths and limitations

This report interrogates the configuration of service delivery and the associated costs accrued to sites in the five service models for children with ID and BtC. It builds on previous research undertaken within the broader programme of work, which had defined service models in order to identify their characteristics. Quantitative approaches to data collection were complemented by qualitative feedback via free text data responses and online meetings, providing insight into service design, configuration and activity. The survey findings were mapped to the data captured from survey 1, to provide contextual relevance to the findings. This research is the first of its kind; an exploratory narrative in the current evidence gap to discover the data that can be collected and how to best interpret it, and to investigate which gaps remain.

Various considerations emerged from this review and are discussed in further detail below.

### Cost estimation for the service models

The objective of this review was to undertake a cost analysis of BtC service models, which involves identifying and measuring the costs of infrastructure, staffing, operations and other service investments adjusted by caseload throughput, to provide early insights into the provision of services for families with BtC. Across the five service models, some data was provided regarding cost and service organisation, however significant data gaps emerged resulting in full or partial cost data missingness, and with insufficient data for meaningful imputation. Full costing for the services was not possible for most of the sites, and operational and overheads costs were almost completely missing. Staffing configuration, a key cost driver of services tended to be fairly well completed, though the data reported by some sites lacked granularity, such as total staff count or full-time equivalence in working hours. There was a lack of information about the heterogeneity of clients and thresholds for eligibility criteria, and sites did not report on their client case-mix throughput across the domains of mild, moderate or severe intellectual disability with BtC. This meant that it was not possible to estimate a generic cost of each service model, adjusted by patient severity.

### Data readiness

It proved extremely challenging to collect the data for this review. Significant effort was invested in reaching out to sites through persistent contact across a year. The work-related demands on frontline staff leaves them with little capacity to respond, exacerbated by a lack of readily available data to complete the survey. Furthermore, services are integrated to the point of not being distinct, and a complexity of this entanglement meant data precision was traded for guesstimates.

Whilst service integration has been a policy directive and optimal service design for BtC was identified as a national priority a decade ago, it is imperative that decisions for funding and staffing arrangements are informed through accurate and current data. Service level data is notoriously difficult to collect and to disaggregate, due to service integration or due to historic legacy organisational or funding arrangements. Yet this data is needed to identify a real-time assessment of community services and supports, population to service planning frameworks, workforce modelling, and persisting service and integration gaps. A minimum dataset of service characteristics that includes funding flows and arrangements, staffing magnitude, case-mix and configuration, jurisdictional integration, population heterogeneity and population reach will enable real-time monitoring for the interventions, treatments and broader changes needed to deliver positive outcomes for families with BtC.

The establishment of routine data capture approaches could be used to investigate service model attributes such as person-centred care, service integration, family support and the professional skills and competencies required. Routine linked data could additionally be used for modelling the parameters of costs and the thresholds for optimal service design.

### Service model jurisdiction and arrangements

The first mapping exercise was undertaken to explore the organisation and provision of BtC services across England ( Taylor *et al.*, 2023). It was able to identify and classify services, although precise mapping of the geographic locations was not undertaken because the service boundaries were unclear. The distribution of aggregate services varied across England; a fifth were in London, the North-East and the North-West; approximately fifteen percent were in the Midlands and the South-East, and with around ten percent in the South-West and the East of England ( Taylor *et al.*, 2023). ‘Population served and reached’ was simultaneously explored in this costing study, but was generally inconsistently completed. Neither survey fully explored jurisdictional and governance structures, such as agreements and standards in a local area that could facilitate a national, whole-of-system framework for consistent and quantifiable services. There is a possibility that a ‘hub and spoke’ model of care operates across services in geographic regions as referrals between sites were reported, but the variability in services implies that a whole-of-system coverage is unlikely. While some sites reported clear eligibility criteria, many did not. The heterogeneity of the service model clientele is likely to significantly impact on service configuration, types of supports offered and key cost-drivers, and it was not possible to disaggregate these with greater granularity.

Many sites reported significantly long waiting lists and delays in treatment provision that allude to significant unmet need. This suggests that the overall BtC service is under-funded and aligns with the findings of Hassiotis *et al.* ( 2020; 2022), that families struggle to identify or enrol their children in local services. Waiting lists for care services have significant implications for human costs in terms of deteriorating health for patients, and provider burnout. Delays in diagnosis and treatment can lead to a worsening of symptoms, which may become more difficult to manage or even irreversible over time. Parents and caregivers bear a significant emotional burden including high levels of psychological distress, including anxiety, helplessness and frustration. Families and caregivers may experience a loss of productivity, lower wages, covering the costs of private healthcare to avoid long waits, and increased reliance on social support systems. Waiting lists also hinder the efficiency of services, creating a backlog in care pathways which prevent new patients from being seen. Ultimately, long waiting lists can exacerbate health inequalities, as more affluent individuals may be able to afford care, while those who rely on public services face prolonged waits ( Brown *et al.*, 2002; Care Quality Commission, 2024; NHS Confederation, 2023; Wisk & Witt, 2012).

Feedback for this report provided by parents and carers revealed a pattern of inconsistency in access to Child and Adolescent Mental Health Services (CAMHS), widely described as a ‘postcode lottery’. Families reported having to navigate complex and opaque referral systems in which eligibility, assessment, and follow-up depend heavily on local availability rather than clinical need. Young people that were initially accepted for review were discharged after a single assessment, before their needs were fully understood. Such early discharges, often justified by incomplete information or narrow eligibility criteria, contributed to disengagement and frustration among families who felt excluded from decision-making. Parents described scenarios in which multiple referral letters were submitted for the same child—sometimes by different professionals—with only one reviewed before discharge. In one illustrative case, five referrals were recorded, but the attending clinician accessed only one. Procedural fragmentation leads to inconsistent clinical judgments, resulting in an emotional toll on families characterised by anxiety, and a sense of being unheard. These experiences point to a need for improved coordination, transparency, and accountability across the referral and triage process.

Difficulties in diagnosis were cited as a source of systemic delay and inequity. Ambiguous presentations across autism spectrum conditions, attention-deficit/hyperactivity disorder (ADHD), and BtC meant diagnostic uncertainty delayed intervention and case ownership. While multidisciplinary team (MDT) working was acknowledged as beneficial, participants noted that MDT functioning was undermined by variability in staffing. Workforce instability was a central limiting factor with services nominally staffed but in practice constrained by long-term vacancies, high sickness absence, and difficulties in recruitment and retention. The shortfall creates a structural mismatch between commissioned service capacity and actual delivery. Families felt they were referred to services that technically exist but were functionally dormant due to unfilled posts. The workforce inadequacy was particularly acute in rural and remote regions, where small health-board areas operated with limited critical mass to provide specialist provision, leading to a geographically uneven system in which the scale and scope of available services differed across health boards and local authorities.

Service fragmentation was also considered to be compounded by the mobility of children and families, especially those requiring out-of-county placements or transitions between education and care settings. Boundary crossings introduced new referral requirements, additional assessments, and renewed waiting times. The cumulative effect resulting in a cycle of delay and discontinuity. Families described being caught between administrative jurisdictions, with services appearing to “work for the system rather than for the family.” Waiting lists for out-of-area CAMHS provision can extend for many months. The needs of CYP with complex communication challenges were flagged as misunderstood or minimised due to communication barriers, and a lack of accessible advocacy mechanisms for these children represents an equity gap.

The participants perceived funding mechanisms to be arbitrary and inconsistent, varying within commissioning bodies. Decision-support tools used to allocate care packages were described as formulaic with funding inconsistencies producing divergent outcomes for similar cases. Inpatient care was widely regarded as an unsuitable solution for most cases, suggesting the need for a funding re-balance towards community-based care including social-prescribing, with wrap-around family support. Such a shift would align financial flows with preventive and person-centred models of care, transforming the current safety net into a coherent continuum of support.

Given that the rationing of health care though waiting lists is pervasive across England, and the integration of services is inconsistent and lacks co-ordination across community services, gaps in the provision of services for BtC are likely to exist, and they are potentially under-reported and persistent. It is therefore also likely that inequities in access to services exist due to misalignment between service types, and levels of intensity and integration when compared to the size and needs of sub-populations ( Brown *et al.*, 2002; Care Quality Commission, 2024; NHS Confederation, 2023; Wisk & Witt, 2012). Future regional commissioning could consider streamlining services across geographic areas through greater co-ordination between sites, and by sign-posting treatment pathway options by patient severity and need.

### A person-centred approach to estimating BtC treatment pathways

The research was limited to the scope of exploring service model organisation and costs through a lens of staffing configuration for service delivery – and it did not extend to capture the full cost funding for CYP with BtC, which would include the costs of CYP care packages. Significant variations in treatment options for CYP with mild, moderate or severe physical or intellectual disability exist which impact on treatment demand, and subsequent costs. The heterogeneity in presentation needs to be central to care planning for appropriate supports; one child may present with a mild form of intellectual disability and BtC, whilst another could perhaps have a genetic condition where the associated phenotype includes being non-verbal with cardiac problems, musculoskeletal difficulties leading to poor exercise ability and tolerance, and be experiencing depression. The majority will be receiving care whilst living in the community with their families, accessing SEND schools as well as a package of care which may involve intermittent carers and short breaks/respite (based on expert consultation in semi-structured interviews with practising clinicians).

While sites did not report cost shifting between services, there is an interplay between social care services, education and health depending on how and where treatment is delivered, in part due to the decision support tools used by local authorities to assess thresholds for healthcare funding. The funding can be allocated to education, health and/or social care budgets at different proportions. A person-centred approach to BtC funding, whilst out of scope here, would capture these broader BtC funding implications. In particular, an approach that captures severity of need would likely alter the costing narrative and would consider the full spectrum of support packages, use of social care services, intensity of care support such as care ratios, judicial reviews of bespoke child care plans, and multi-disciplinary team oversight. Children with severe physical and intellectual disabilities with BtC may also cycle between social services such as child in need (CIN), child protection orders (CPN), and dynamic support registers in CAMHS settings and health care (based on expert consultation in semi-structured interviews with practising clinicians).

Local authorities will use less of some services and more of others, by level of need and by the current availability of services. Reviewing case-mix within sites as well as across sites will also enable commissioners to explore optimal service configurations. ( The Kings Fund, 2023). A stepped model of care may provide best practice treatment pathways in some contexts, whereas front loading senior clinicians for diagnosis, staff supervision and safeguarding in a more comprehensive intensive model of care may better suit others. Efficiencies within some services may improve with an approach favouring episodic ‘acute’ care management to include discharge, as opposed to ongoing psychological support and continuity of care. Yet approaches for service design need to account for the developmental needs of a child, which may change during adolescence, thus a person-centred approach to funding investment, would enable an evaluation of cost-saving models of care. An example is the development of the "Ealing Model of Care" for CYP with complex needs, which also includes the multi-agency collaboration, the “Ealing Brighter Futures Intensive Engagement Model” ( Munro *et al.*, 2017). The design of an Ealing person-centred service-model led to the identification of key priorities that included early intervention, family-centred engagement with personalised care planning, and intensive support for complex needs. Such an approach might be considered ‘invest to save’, where the cost of investment in an intervention in the immediate period accrues benefits (measured by changes in outcomes) in the longer term or across the lifespan.

### Estimating outcomes that inform cost-effectiveness of service model design

A potential approach would extend beyond a cost analysis or ROI, to compare the cost of an intervention to the health or social benefit gained, considering factors beyond monetary value. Evaluating services for BtC for future cost-effectiveness analysis requires data on costs and outcomes. Whilst not in scope for this review; measuring and valuing the changes in outcomes for the children and young people (and their families) would enable services and healthcare providers to assess the effectiveness of their treatments, make informed decisions about treatment plans, track progress over time, and identify areas for improvement for well-being and quality of life. Validated instruments have been developed to estimate the changes in both the quantity and quality of health-related quality of life gained. The Child Health Utility 9 Dimensions (CHU-9D), is a paediatric generic preference-based measure of health-related quality of life is suitable for 7 to 17-year olds ( Stevens, 2010). Another instrument, the Paediatric Quality of Life Inventory is validated to explore physical, emotional, social and school function for CYP from two years upwards ( Varni *et al.*, 1999). A third instrument, the EuroQol Five Dimensions for Youth and Three Levels (EQ-5D-Y-3L) comprises the following five dimensions of mobility, self-care, usual activities, pain/discomfort and anxiety/depression ( Devlin *et al.*, 2020).

There are also validated instruments that measure outcomes for children with disabilities. The Cerebral Palsy Quality of Life Questionnaire (CP QoL-Child) assesses quality of life in children with cerebral palsy (CP) aged between four and twelve years of age ( Chen *et al.*, 2013). The Generic Children's Quality of Life Measure (GCQ) measures quality of life in children aged between six and fourteen and was developed to allow comparison between with the general population, for children with specific health or social difficulties, especially chronically ill children ( Collier *et al.*, 2000). The KIDSCREEN tool is a validated instrument for measuring health related quality of life in children and adolescents aged between eight and eighteen, measuring physical well-being, psychological well-being, autonomy and parent relations, peers and social support, and the school environment ( Ravens-Sieberer *et al.*, 2010). Finally, the Pediatric Quality of Life Inventory Duchenne Muscular dystrophy (PedsQL DMD) has been designed to evaluate for quality of life outcomes in children and adolescents with neuromuscular diseases ( Uzark *et al.*, 2012). A systematic review of multidimensional childhood patient-reported outcome measures summarises all the generic non-preference based and preference-based measures, their characteristics and where relevant, their accompanying value sets provides comprehensive insight into approaches to estimate Health Related Quality of Life for the child ( Kwon *et al.*, 2022).

Extending outcomes measurement to families would enable data to be captured for parents and children. Parental health-related quality of life outcomes can be collected separately using the EQ-5D-5L. It is a multi-attribute generic instrument and it has been translated into more than 150 languages and is recommended by the Health Technology Assessment (HTA) bodies such as the National Institute of Health for Health and Care Excellence (NICE). Another widely used, generic patient-reported outcome measure (PROM) of health-related quality of life is the 12-Item Short-Form Health Survey (SF-12 ( Walters & Brazier, 2003)). It has eight dimensions, which explore limitations in physical activities, physical or emotional problems, and /or usual role activities because of physical or mental health problems. More broadly, it also considers bodily pain, general mental health, vitality and health perceptions.

It is anticipated that quality-adjusted life year (QALY)-based approaches are not yet sufficiently developed to capture all the effects of BtC interventions for both parents and children together, due to methodological challenges surrounding aggregation of disparate benefits for parents and children in a single metric. To combine disparate outcomes for parents and children in a single preference-based outcome measure would be a methodological opportunity for a future economic evaluation.

### Recommendations for commissioners

While service model configuration and costs have been explored in this research, the following issues should be considered when contemplating BtC service-model funding and investment, which would also require a thoughtful application to local and regional context.

- Consider collecting a minimum dataset of current services. This could include service characteristics of funding flows and arrangements, staffing magnitude, case-mix and configuration, jurisdictional integration, service coverage and patient heterogeneity, in particular across mild, moderate and severe BtC.
- Consider mapping primary service and specialist service gaps, to support the streamlining of services across geographic areas through greater co-ordination between sites, and by sign-posting treatment pathway options by patient severity and need.
- Consider the design of a person-centred service-model prioritising early intervention, family-centred engagement with personalised care planning, and intensive support for complex needs.
- Capture longer term changes in outcomes for CYP with BtC and their families, which will inform treatment effectiveness and feed into cost-effectiveness analyses for models of care.

Measuring and valuing the changes in outcomes for the children and young people (and their families) treated by the services would enable healthcare providers to assess the effectiveness of their treatments, make informed decisions about treatment plans, track progress over time, and identify areas for improvement for well-being and quality of life. A key message from this research is that there seems to be little oversight generally of what is delivered for BtC, how, or how effective it is.

### Implications for future research

This is the first-time research of this nature has been attempted with these types of services, meaning there is an exploratory element to the work wherein we are considering what type of data it is possible to collect and what can be interpreted from the data. Further research aims to explore the connection between outcomes for children and young people, their families and service models; within MELD another study is underway to estimate the cost-effectiveness and cost-consequences following a referral into a specialist BtC service compared to other models of services. The health economics data will be gathered from individual families to complement the top-down approach in this report – because the data will be more varied in terms of the services and supports that different families receive from the same service. The cost-effectiveness analysis aims to estimate the incremental cost per reduction in behaviour problems, as measured by the Behaviour Problems Inventory Short Form. (BPI-S) (Total Score) ( Rojahn *et al.*, 2012). The BPI-S is a carer-reported behaviour rating instrument for intellectual disabilities with three sub-scales: self-injurious behaviour, stereotyped behaviour and aggressive/destructive behaviour. Cost-consequences analyses will additionally be undertaken, as they are recommended for complex interventions that may have multiple implications ( Drummond *et al.*, 2005), and for public health interventions which may have an array of benefits that are difficult to synthesise in a common unit ( NICE, 2013). They present disaggregated costs and disaggregated consequences (primary and secondary outcomes) in a disaggregated form, together with the estimates of the mean costs of the comparator interventions with appropriate measures of dispersion.

Broader resource consequences and associated economic costs will be informed by Client Service Receipt Inventory (CSRI) completed by each child’s primary parental caregiver at baseline and at a 12-month follow-up. These will consider health care costs to families and carers (e.g. medication, community health care, inpatient or outpatient care) and broader costs to families (e.g. time off work, time spent travelling to appointments). Data items will be captured in disaggregated units where possible, and micro-costing will be performed to capture variance in costing patterns.

The cost-consequences analysis will derive incremental values for all relevant categories of secondary outcomes including child-related outcomes, family-carer-related outcomes, service-level outcomes and other stakeholder-related outcomes. Child-related outcomes, will be completed by family carers and will include child quality of life measures including EQ-5D-Y ( Wille *et al.*, 2011) and the Paediatric Quality of Life Inventory (PedsQL) ( Varni *et al.*, 2011). Family-carer-related outcomes will use the EQ-5D-5L Health Questionnaire ( van Hout *et al.*, 2012) for changes in quality of life. It is anticipated that this additional research will address some of the evidence gaps that have emerged from this ‘top down approach’ to the costing of the service models, and the costing data obtained here will feed into the future evaluation.

## Study registration

Trial Registration: Current Controlled Trials ISRCTN88920546, Date assigned 05/07/2022.

## Ethics declarations

Ethics approval and consent to participate

All methods were performed in accordance with the relevant guidelines and regulations. This study received approval from the Humanities and Social Sciences Research Ethics Committee at the University of Warwick (REF: HSSREC 91/20-21). Informed consent was obtained from all participants and participating organisations.

This research complies with the Declaration of Helsinki: https://www.wma.net/policies-post/wma-declaration-ofhelsinki/

## Data Availability

The data underlying this study were provided in confidence by participating local authorities and contain commercially and operationally sensitive financial information relating to staffing, service overheads, and cost structures. The specialist nature of the services and small sample of sites means there is a high risk of identification of particular services in the study were the data to be provided at a level with any more detail than is currently in the manuscript. ***- a description of the restrictions on the data;*** These data were accessed under strict data-sharing agreements that prohibit onward sharing or public release of either raw or aggregate figures beyond those reported in this article. The datasets therefore cannot be made publicly available for reasons of organisational confidentiality and data-protection obligations. All analyses presented in the paper are based on anonymised and aggregated data supplied under these agreements. Only the summary statistics and findings reported in the article may be shared publicly. ***- the relevant Institutional Review Board (IRB)*** This study’s research programme and data collection methodology received approval from the Humanities and Social Sciences Research Ethics Committee at the University of Warwick (REF: HSSREC 91/20-21). ***- necessary information required for a reader or reviewer to apply for access to the data and the conditions under which access will be granted.*** Researchers who require further information about the study design or analytic approach may contact the corresponding author for clarification, but access to the original data would require new data-sharing agreements with the contributing local authorities. Extended data associated with the manuscript “Community-Based Service Models for Children with Intellectual Disabilities and Behaviours that Challenge (BtC) in England: Configuration, Costs and Evidence Gaps”, undertaken as part of the Mapping and Evaluating Services for Children with Learning Disabilities and Behaviours that Challenge (MELD) programme, are available as “MELD OSF Extended Data”. These data are underlying the results reported in this article and are available via the Open Science Framework (OSF). The extended data comprise the survey instrument, unit cost data, and a data completion summary for the Report. The data are available under a Creative Commons CC0 1.0 Universal licence. Data can be accessed at: https://doi.org/10.17605/OSF.IO/CJWBM
