# Supplementary material for: The configuration, funding and costs of providing services for children with behaviours that challenge. A report undertaken as part of the Mapping and Evaluating Services for Children with Learning Disabilities and Behaviours that Challenge (MELD)
Source: NIHR Open Res. 2026 Feb 11;6:13. [Version 1] doi: 10.3310/nihropenres.14153.1 (PMC13044538; doi:10.3310/nihropenres.14153.1)
Supplement: Peer review history

# Reviewer report: Reviewer response for version 1

**DOI:** 10.3310/nihropenres.15400.r39855

**Published:** 2026-04-01

**Authors:** Kerim Munir

**Affiliations:** 1 Boston Children's Hospital ,Harvard Medical School, Boston, Massachusetts, USA, Boston, Massachusetts, USA

Overview:

 This is an important policy-relevant paper addressing a major service gap for children with intellectual disabilities and behaviours that challenge (BtC) in England.

 The paper attempts to move beyond broad policy discussion and describe how community services are configured, funded, and costed across five service models: (1) Child and Adolescent Mental Health Services (CAMHS) (43%); ID CAMHS (17%); Children/Young People Disability (16%); Specialist BtC Support (17%); and All age (7%). It makes a useful contribution, particularly because the authors situate the work within the context of Transforming Care, NICE guidance, and current efforts to reduce inappropriate institutional care and strengthen community provision.

 The principal strengths are the project’s national scope, its attempt to link typology and costing, its inclusion of Parent Advisory Group input, and its honest acknowledgment that routine service-level costing data are very difficult to obtain in real-world systems. The paper is especially valuable as a description of the present state of data availability and fragmentation in this field.

 A major recommendation is for authors to more clearly frame the paper as an exploratory, service-mapping study rather than a robust comparative costing analysis. The central concern is not the importance of the question, but the degree of missingness and heterogeneity in the cost data, which substantially limit the strength of the inferences. For example, the manuscript itself states that 278 services were identified; 161 informed the typology; 19 sites were selected for detailed costing; and only 10 sites returned some data. In its current form, the manuscript risks overstating what can legitimately be concluded from the available data. Because the detailed costing phase yielded responses from only 10 of 19 sites, with substantial missingness within and across sections, very limited overhead data, and inconsistent full-time equivalent (FTE) posts devoted to the service being costed reporting. Given the lack of meaningful case-mix adjustment by severity, the paper should be framed more explicitly as an exploratory scoping or feasibility study of service models and data readiness, rather than as a robust comparative costing analysis.

 Recommendations:

1. The paper should be reframed more explicitly as exploratory and oriented toward feasibility. The strongest contribution is not that it provides definitive comparative costs of five service models, but that it shows how difficult such costing currently is in this service landscape. The manuscript itself acknowledges that full costing was not possible for most sites, that operational and overhead costs were almost entirely missing, and that case-mix by severity was unavailable. Under those circumstances, conclusions about service-model optimisation and cost structure should be substantially softened. I would encourage the authors to state earlier and more consistently that this is a top-down, exploratory mapping and costing exercise whose main output is the identification of evidence and data gaps.
2. The sampling flow and analytic denominators need a clearer presentation. The manuscript reports on several denominators: 278 potentially eligible services, 161 respondents to the typology survey, 19 sites selected for detailed costing, and 10 sites that returned some follow-up data. This is important but currently difficult to follow. A flow diagram is needed. The authors should also explain more clearly how the 19 sites were selected as “representative,” how representativeness was judged, whether there were systematic differences between responders and non-responders in the costing phase, and what implications this has for inference. Without this, the reader cannot judge how far these site-level costing observations can be generalized.
3. The transition from Latent Class Analysis (LCA) to five final service models requires more methodological transparency. The paper states that LCA identified two service models, and that descriptive analysis, broader survey, and interview data were used to inform the final classification into five models. That may be entirely reasonable, but the process is not adequately explained. The authors should spell out which variables entered the LCA, how the number of classes was chosen, why the LCA yielded only two classes, and by what explicit decision rules the final five-model typology was produced. At present, the process appears to be partly statistical and partly interpretive, but the balance between them is unclear.
4. The manuscript needs a clearer separation between Stage 1 and Stage 2 findings throughout the Results section. The paper repeatedly combines detailed follow-up costing observations from a very small number of sites with broader descriptive observations from Stage 1. This is sensible in context, but it is not always clear which claims derive from the larger mapping dataset and which from the sparse costing dataset. The phrase that the Survey 2 data were “triaged with” the Stage 1 data is also not sufficiently clear methodologically. The Results would be stronger if each section were explicitly distinguished: (a) findings from Stage 1 mapping data; (b) findings from Stage 2 costing returns; and (c) interpretive synthesis across both.
5. The costing tables will benefit from a revision to improve precision. This is a central issue. The tables present apparently ‘precise’ cost totals. Still, many of these are based on incomplete salary/FTE information, national band-based estimates from the PSSRU compendium, and a “pragmatic” apportionment of overheads when real overhead data were unavailable. In several instances, the paper itself notes that direct comparisons are not possible due to missing FTE data. I think the tables should visually distinguish: (a) observed salary/cost data provided directly by sites; (b) band-derived or benchmark estimates; (c) assumed or imputed components; and (d) totals not suitable for direct comparison. Otherwise, the reader may over-interpret these totals as actual comparable costs rather than partial modelled estimates. The authors should consider presenting these as illustrative resource profiles or indicative cost ranges rather than precise comparative service costs.
6. Case-mix and severity are insufficiently developed for the conclusions drawn. The paper correctly notes that service populations are heterogeneous and that the study could not adjust for mild, moderate, or severe intellectual disability, nor for the severity or the behaviors that challenge. This is an important limitation. It means that one cannot make strong claims about whether one service model is more or less costly or efficient than another, because the populations served may differ substantially in complexity. This limitation should be moved earlier in the paper and explicitly incorporated into the abstract and conclusions.
7. The paper often argues that long waiting lists suggest underfunding, unmet need, service inequity, or structural inadequacy. These are plausible interpretations and may well be correct, but the study design is descriptive and does not directly demonstrate causation. Given the sparse and incomplete cost data, I would encourage the authors to use more cautious language. For example, “are consistent with,” “suggest,” or “raise concern about,” rather than more definitive causal statements. The same applies to broader claims about optimal service design and the implications for future commissioning.
8. Discussion includes detailed statements about parent experiences, postcode lottery effects, early discharge after a single assessment, fragmentation across jurisdictions, workforce dormancy due to vacancies, and inequity in rural and remote regions. These are important observations, but the reader needs to know more precisely where they came from. Were these findings from the Parent Advisory Group, free-text survey responses, online meetings, a distinct qualitative component, or a synthesis of stakeholder consultation? The methods section currently does not support that level of specificity in the Discussion.
9. Terminology should be standardized: The manuscript says it will use “intellectual disability” consistently, but “learning disability” still appears, especially in the MELD programme title and in the Discussion of guidelines and services. Some of this is unavoidable in UK service naming, but the manuscript should distinguish between retained formal service titles and the authors’ preferred descriptive terminology.
10. Some additional minor suggestions to strengthen clarity and consistency include:  a) The title should explicitly mention both intellectual disability and England. As written, it sounds broader than the actual study. b) Tables need all abbreviations to be defined beneath each table, including FTE, WTE, ICB, CCG, LD, CAMHS, BtC, BCBA, PBS, and CTPLD. It should also be clearly stated whether “Salary” refers to the annual salary and whether the adjusted totals include on-costs, qualifications, management overheads, capital overheads, and travel. The tables currently require too much back-and-forth with the text. c) There appear to be typographic and internal consistency issues in the tables. Examples include spacing and punctuation inconsistencies in cost figures, unclear labels such as “Total FTE N/A,” and what appears to be an implausible “assumed 0.5% FTE” for the external supervisor, which may in fact mean 0.5 FTE. These should be carefully checked. d) The phrase “section seven provided free text for miscellaneous information” should be clarified. It would be better to say what kinds of information were sought there, for example, unusual service features, contextual information, non-standard staffing arrangements, or uncaptured costs. e) Service model descriptions could be made much easier to understand. A summary figure or one-page comparison table would help, with columns for each model and rows for eligibility, age range, core disciplines, typical interventions, funding basis, waitlist profile, and data completeness. The current narrative is rich but dense. f) The exclusion of special school services should be justified more explicitly. Given the role schools often play in supporting children with challenging behaviours, the rationale for exclusion should be clearer. g) The phrase “first of its kind” should be softened: A more defendable phrasing would be “the first national exploratory overview” or “the first national top-down service-model and costing exercise of which we are aware” for community-based services in England for children with intellectual disability and behaviours that challenge. h) “Recommendations for commissioners” may be too strong. Given the exploratory nature of the work and the acknowledged missingness, “Implications for commissioners” or “Considerations for commissioners” would be more appropriate than formal recommendations.

Implications for future research:

 I would recommend adding the following closing comment: This policy-relevant paper addresses an understudied yet highly important area. Its value lies primarily in national service mapping, early refinement of typology, and candid exposure of the current limitations of service-level costing data. With revision, the paper stands to make a strong contribution as an exploratory study of service configuration and data readiness. To achieve that, however, the authors should narrow some of their claims, improve methodological transparency, more clearly separate Stage 1 from Stage 2 findings, and revise the costing tables to reduce false precision and improve interpretability.

Is the work clearly and accurately presented and does it cite the current literature?

Partly

If applicable, is the statistical analysis and its interpretation appropriate?

Partly

Are all the source data underlying the results available to ensure full reproducibility?

Partly

Is the study design appropriate and is the work technically sound?

Partly

Are the conclusions drawn adequately supported by the results?

Partly

Are sufficient details of methods and analysis provided to allow replication by others?

Yes

Reviewer Expertise:

I am a Professor of Pediatrics and Psychiatry with extensive experience in intellectual and neurodevelopmental disorders, and I have served in Leadership Education in Neurodevelopmental Disorders and Related Disabilities (LEND) in the United States. I serve as Chair of the World Psychiatric Association Scientific Section in Psychiatry of Intellectual and Developmental Disorders. My ORCID No. is 0000-000202404-1806

I confirm that I have read this submission and believe that I have an appropriate level of expertise to confirm that it is of an acceptable scientific standard, however I have significant reservations, as outlined above.

# Reviewer report: Reviewer response for version 1

**DOI:** 10.3310/nihropenres.15400.r39849

**Published:** 2026-03-24

**Authors:** Laurence Taggart

**Affiliations:** 1 Queens University Belfast, Belfast, Ireland

This is a timely and important paper, given the increasing policy and service emphasis on supporting children and young people with intellectual disability who present with behaviours that challenge, and the associated demands placed on families and services. The study addresses a clear gap in understanding service configurations and associated costs in England.

 I have a small number of minor suggestions to strengthen clarity and consistency:

**Title and Abstract**

- The title should explicitly state that the study focuses on children with intellectual disability and was conducted in England
- Please ensure consistent use of the term intellectual disability throughout, rather than learning disability
- Avoid abbreviations where possible; terms such as behaviours that challenge and intellectual disability should be written in full

**Background**

- This section is well written and provides a strong overview of the current legislative and policy context in England.
- In describing the five service models, it would strengthen the paper to 1) Specify the professional groups involved in each model (noting that this is only partially described, e.g., nursing in Model 2) Outline the types of interventions/therapies delivered within each model (e.g., behavioural and psychological support is only explicitly referenced in Model 4).
- Ethical approval was obtained

**Data Collection**

- Clarify that Stage 1 survey findings have been reported elsewhere.
- This paper reports Stage 2 (follow-up survey and interviews), which aimed to collect more detailed data on staffing, overheads, and cost structures.
- Nineteen sites participated in this phase but only ten responded.
- The purpose-designed data collection instrument (seven sections) completed by clinical leads/team managers and finance staff is appropriate
- The involvement of a PPI group (including parents, children and young people, the Challenging Behaviour Foundation, and a Parent Advisory Group) are a strength.

**Results**

- Data completeness is a key limitation: only 10/19 sites returned data, and only two sites per model provided complete datasets. This is appropriately acknowledged
- Funding structures and flows are clearly described and well supported with tables
- Operational and overhead costs are less complete; Models 2 and 5 provided no data, and other models provided limited information
- Staffing data are clearly presented, demonstrating substantial variation in professional composition across sites and models
- Reported staff costs vary and appear sensitive to contractual arrangements, agency use, and on-call requirements
- The inclusion of staffing organograms and associated cost tables is useful; however, interpretation should be cautious given the extent of missing and inconsistently reported data
- The description of service configuration and community reach across models is informative

**Discussion**

- This is a novel and valuable contribution, providing insight into the configuration and costs of service models for this population in England
- Limitations are clearly acknowledged, particularly the extent of missing cost data and the inability to undertake robust or complete economic analyses
- Full costings were not possible for most sites, and overhead/operational cost data were largely unavailable for some
- Site engagement was limited (10/19), which may affect representativeness
- The recommendations for commissioners and future research are appropriate and helpful

Overall, this paper makes a strong and valuable contribution to an under-researched but policy-relevant area. Its key strength lies in attempting to systematically characterise and compare service models and associated costs for children and young people with intellectual disability and behaviours that challenge, an area where empirical evidence is notably limited. The inclusion of multiple sites, engagement with stakeholders including PPI contributors, and the transparent reporting of challenges in data completeness all enhance the credibility and usefulness of the work. Despite limitations in the dataset, the study provides important preliminary insights that can inform commissioners, support service planning, and lay the groundwork for more robust future economic and implementation research in this field.

Is the work clearly and accurately presented and does it cite the current literature?

Yes

If applicable, is the statistical analysis and its interpretation appropriate?

Yes

Are all the source data underlying the results available to ensure full reproducibility?

Yes

Is the study design appropriate and is the work technically sound?

Yes

Are the conclusions drawn adequately supported by the results?

Yes

Are sufficient details of methods and analysis provided to allow replication by others?

Yes

Reviewer Expertise:

Intellectual disability research, implementation science

I confirm that I have read this submission and believe that I have an appropriate level of expertise to confirm that it is of an acceptable scientific standard.

## References

[1] : The configuration, funding and costs of providing services for children with behaviours that challenge. *NIHR Open Research, 6:13, https://doi.org/10.3310/nihropenres.14153.1* .2026;10.3310/nihropenres.14153.1PMC1304453841939691

# Reviewer report: Reviewer response for version 1

**DOI:** 10.3310/nihropenres.15400.r39852

**Published:** 2026-03-20

**Authors:** Sunil Karande

**Affiliations:** 1 Pediatrics, Seth GS Medical College and KEM Hospital, Mumbai, India, Mumbai, Maharashtra, India

In England there is now a national policy approach to shift care for people with intellectual disabilities and autism from hospitals to community settings. Keeping this national policy in mind the Authors have attempted to document the ground realities of implementation of this policy. The Authors have written an extensive and detailed manuscript which explores the structure and associated costs of community-based services in England that support children with intellectual disabilities and behaviors that challenge (BtC). The Authors have made a sincere attempt to describe the configuration of local “service models”, identify cost drivers, and clarify data requirements for commissioning and evaluation. The Authors faced lots of problems in getting the data and many a times the data was inadequate. I applaud the Authors efforts to venture onto such a complex research objective. Not surprisingly the Authors, at the end of their data collection and analysis, found critical and persistent gaps between policy goals and the reality of service delivery. These are my comments which are meant to help the Authors improve their manuscript:

 1) Addition of a flow diagram showing the objectives, displaying that 278 services were identified across England and only 161 services participated in the study. The flow diagram can show the five distinct service models identified and under each service model the most important findings of the present study should be stated. This flow diagram would improve the readability of the manuscript.

 2) The Authors should clarify why attempts to complete collection of the data were not made by actually visiting these services after getting the incomplete data by email or over the phone.

 3) Why was the service excluded if it was a special school service? Please elaborate the reason for this exclusion criteria.

 4) The Authors should add a brief paragraph about the care given to children with intellectual disabilities and BtC in the areas in England where the community based services are not yet functional or are still not optimally functional. The Authors should clarify whether the old system of hospital based treatment is still being followed or it has been completely stopped.

 5) The Authors have stated that 'section seven provided free text for miscellaneous information,

 to inform a comprehensive understanding of the variation in key service components, items or costs". I request the Authors to simplify this sentence and clarify what is being meant by 'miscellaneous information'.

 6) The Authors have stated that "Model 5 - All age:

 No sites reported data for overheads and operational costs". This reinforces my earlier comment that a personal visit by one of the Authors would surely have helped in documenting this data. This is a limitation of the present study and it may not be due to less than optimal implementation of the national policy.

 6) The Authors should improve the Tables. In general the information displayed in a Table should be understood by a reader without reading the text. Accordingly, I request the Authors to give the full form of each abbreviated word in the Tables; for example FTE, WTE, Salary (is it per month or yearly), give a reference for how the pay grade is decided. This can be stated below the Table in a smaller font size.

 7) The reader should be able to understand the difference between the term Vulnerabilities

 Rotational Nurse, LD Nurse and Associate Nurse. 

 8) The Authors should not claim that "This research is the first of its kind; an exploratory narrative in the current evidence gap to discover the data that can be collected and how to best interpret it, and to investigate which gaps remain". At best the present manuscript is a pilot research study. To get all the relevant data as stated in the objectives of the present study - the Authors should have had a more thorough methodology in collecting the data; they should have interviewed parents of afflicted children, got the costs of running the service with stronger efforts, etc.

 9) In my opinion, the Authors should change the term: "Recommendations for commissioners" to "Suggestions for commissioners". The methodology of the present study is not that robust to justify the use of the word "recommendations".

Is the work clearly and accurately presented and does it cite the current literature?

Yes

If applicable, is the statistical analysis and its interpretation appropriate?

I cannot comment. A qualified statistician is required.

Are all the source data underlying the results available to ensure full reproducibility?

Partly

Is the study design appropriate and is the work technically sound?

Partly

Are the conclusions drawn adequately supported by the results?

Partly

Are sufficient details of methods and analysis provided to allow replication by others?

Partly

Reviewer Expertise:

I have recently retired as a Professor of Pediatrics and Head of Department. I have during my 32 years career as a Medical Teacher, Consultant and Researcher closely delt with children with intellectual disabilities and BtC. My ORCID No. is 0000-0002-1170-325X and I am unable to connect to ORCID from this website.

I confirm that I have read this submission and believe that I have an appropriate level of expertise to confirm that it is of an acceptable scientific standard, however I have significant reservations, as outlined above.

## References

[1] : The configuration, funding and costs of providing services for children with behaviours that challenge. *NIHR Open Research* .2026;6: 10.3310/nihropenres.14153.1 10.3310/nihropenres.14153.1 PMC1304453841939691
